# Osmotic Force Balance Evaluation of Aqueous Electrolyte Osmotic Pressures and Chemical Potentials

**DOI:** 10.1021/acs.jctc.3c00982

**Published:** 2023-11-18

**Authors:** Alireza Hosseni, Henry S. Ashbaugh

**Affiliations:** Department of Chemical and Biomolecular Engineering, Tulane University, New Orleans, Louisiana 70118, United States

## Abstract

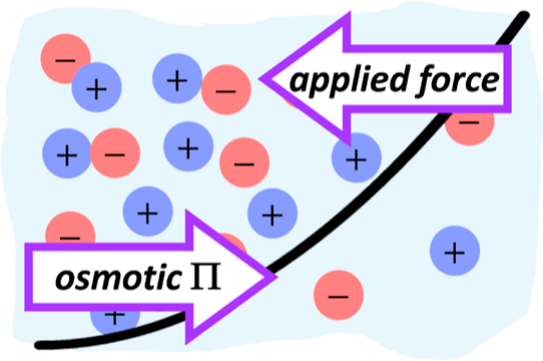

Concentrated aqueous salt solutions are ubiquitous in problems of biological and environmental relevance. The development of accurate force fields that capture the interactions between dissolved species in solution is crucial to simulating these systems to gain molecular insights into the underlying processes under saline conditions. The osmotic pressure is a relatively simple thermodynamic property connecting the experimental and simulation measurements of the associative properties of the ions in solution. Milner [C. Gillespie and S. T. Milner, *Soft Matter*, **16,** 9816 (2020)] proposed a simulation approach to evaluate the osmotic pressures of salts in solution by applying a restraint potential to the ions alone in solution and determining the resulting pressure required to balance that potential, referred to here as the osmotic force balance. Here, we expand Milner’s approach, demonstrating that the chemical potentials of the salts in solution as a function of concentration can be fitted to the concentration profiles determined from simulation, additionally providing an analytical expression for the osmotic pressure. This approach is used to determine the osmotic pressures of 15 alkali halide salts in water from simulations. The cross interactions between cations and anions in solution are subsequently optimized to capture their experimental osmotic pressures.

## Introduction

Aqueous electrolytes play a vital role in biological processes and in environmental waste remediation. Potassium^1^ and sodium^2^ channel proteins, for instance, regulate muscle and neuron activity, utilizing selectivity filters to promote the flow of specific cations across cell membranes.^3^ In bacterial colonies, on the other hand, ion channels conduct long-range electrical signals across biofilms to coordinate metabolic states among the cells.^4^ Multivalent metal ions like Ca^2+^ and Zn^2+^ act to moderate processes including cell fusion,^5^ apoptosis,^6^ enzymatic activity,^7^ and protein binding to nucleic acids and other proteins.^8^ On the macro-scale, osmoreceptor nerve cells help regulate the osmotic balance in the body by moderating the intake and discharge of water and salt via thirst and renal excretion.^9^ While molecular simulations are extensively used to gain molecular insights into biological functions,^10^ the development of intermolecular potentials to describe ion interactions under physiological conditions is an area of intense research as a result of the challenges of using classical force fields to capture specific ion effects.^11−14^

The osmotic pressure of ions provides the impetus for flow across semipermeable membranes. In the reverse osmotic purification of water from salt solutions, the pressure of a waste effluent stream must be increased so that the pressure difference between the effluent and purified water is greater than the osmotic pressure. If the pressure drops below this threshold, water can flow back to the effluent. The growth in the treatment of produced water, water extracted from underground formations as a byproduct of oil and gas production,^15^ for either reuse in oil and gas extraction or for beneficial use (e.g., agriculture) is expected to outpace reinjection for the foreseeable future, accounting for more than half of the $10 billion water management market by 2025.^16^ Membrane separations have received significant attention for produced water treatment due to the fact they can produce higher-quality water via a less chemically intensive process while being cost competitive with alternate strategies.^17,18^ Salts comprise one of the dominant constituents of produced water.^15^ As such, a better understanding of the osmotic properties of salts in solution can beneficially impact the design of water purification systems. For example, simulations have shown that the pressure required to affect water flow through membranes against the osmotic pressure of aqueous NaCl solutions is significantly underestimated if the salt is assumed to obey the ideal gas law.^19^

The development of molecular simulation protocols and models to accurately capture the osmotic properties of salt solutions is an active area of ongoing research.^20^ Perhaps the earliest simulation approach for the evaluation of the osmotic pressure from molecular simulations was proposed by Murad and Powles.^21^ In their method, two semipermeable membranes are used to partition the simulation box into pure solvent and mixture cells. The osmotic pressure is determined by the pressure difference between the mixture and solvent cells. Luo and Roux^22^ later adapted this technique to examine the osmotic equation-of-state of aqueous electrolyte solutions. Rather than model an explicit membrane, which can introduce solution structuring that can muddle interpretation of finite-sized simulations,^23^ they introduced half-harmonic potentials to act as virtual walls that act only on the ions but not the water. The osmotic pressure is determined by the force of the ions on the walls. Kohns et al.^24^ used Luo and Roux’s approach to evaluate the activity of water in NaCl solution. Saxena and García,^25^ on the other hand, used this approach to reparametrize divalent ion interactions to minimize clustering in the simulation to bring the osmotic pressure into agreement with experiment. Alternately, the osmotic pressure was evaluated from the free energies of various components in aqueous electrolyte solutions. The osmotic pressure is evaluated from these calculations as the pressure required to raise the chemical potential of water in the mixture to that of pure water at atmospheric pressure. Benavides et al.,^26^ for example, used thermodynamic integration to evaluate the free energies of NaCl in water, from which the chemical potential of water and osmotic pressure can be inferred. Nezbeda and co-workers,^27,28^ on the other hand, used a combination of open ensemble and isothermal–isobaric ensemble simulation techniques to evaluate a wide range of solution properties of aqueous NaCl solutions including its osmotic pressure.

A difficulty with the techniques described above is that multiple simulations must be performed to examine the concentration dependence of the osmotic equation-of-state. Moreover, following the free energy simulation approaches, the osmotic pressure frequently is only indirectly inferred after processing of results for the chemical potentials of the ions and water in the mixture. Milner^23^ recently proposed a simulation technique that circumvents these difficulties, referred to here as the osmotic force balance. In the osmotic force balance, an external potential acting along a single axis of the simulation box is applied to the solutes but not the solvent. This external potential is balanced by the osmotic pressure gradient that results from the nonuniform distribution of solutes in the simulation box. Milner and co-workers used this approach to evaluate the osmotic pressure of NaCl,^23^ poly(ethylene oxide)^29^ in water, and mixtures of benzene and pyridine.^30^ The osmotic force balance is an extension of the sedimentation equilibrium approach proposed by Hansen and co-workers^31,32^ to evaluate the equation-of-state of colloidal particles. In these simulations, an effective gravitational field is applied to the particles in solution, giving rise to a concentration gradient in the simulation. Hansen’s approach has been used in simulations and experiments to determine the equation-of-state of hard spheres,^31,32^ charged colloids,^32,33^ Janus colloids,^34,35^ granular suspensions,^36^ and supercooled water.^37^ The main difference between the Milner and Hansen approaches is that Hansen considered one-component systems, while Milner selectively applied the external potential to individual components of a mixture to establish osmotic equilibrium.

Here, we expand the osmotic force balance method to determine the free energies of aqueous electrolytes over a range of concentrations from a single molecular simulation. We also explore the connection between calculations performed with the chemical potential of water fixed and calculations performed with the bulk pressure fixed to obtain an improved comparison between simulations and experiment. The methods developed here are then applied to simulations of 15 alkali halide salts in aqueous solution (excluding the fluoride salts) to evaluate their osmotic pressure, activity coefficients, and solution density. The Lennard-Jones interactions between cation–anion pairs are then optimized so that the simulations reproduce the experimental osmotic equations-of-state for each salt.

## Theory

### Osmotic Force Balance

Consider an electroneutral system of solutes (e.g., ions) in solution with an external potential applied to the solutes along the *z*-axis, *U*(*z*) but not to the solvent. Here, we assume that this potential is zero at the origin (i.e., *U*(0) = 0 at *z* = 0) and monotonically increases with distance from the origin. As such, the solutes will congregate near the minimum in the applied potential. The variation in the solute concentration subsequently establishes a gradient in the osmotic pressure, Π(*z*). Millner^23^ demonstrated that the osmotic pressure at equilibrium satisfies the force balance
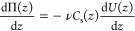
1where the left-hand side corresponds to the gradient of the osmotic pressure and the right-hand side is the opposing force acting on the solutes by the applied field. In this expression, *C*_s_(*z*) is the solute concentration at *z*, while ν is one for a neutral solute and is equal to the sum of the stoichiometric coefficients for a salt, e.g., ν = ν_+_ + ν_–_ for salt *X*_ν+_*Y*_ν–_. As an aside, *C*_s_ is determined as *N*_s_/*V*, where *N*_s_ is the number of solute molecules and *V* is the volume. The number of cations and anions in solution are *N*_+_ = ν_+_*N*_s_ and *N*_–_ = ν_–_*N*_s_, respectively. This expression reduces to that utilized by Hansen and co-workers^31,32^ when the solute concentration is replaced by that of the single component in the system and the potential gradient term is replaced by the gravitational acceleration constant. The osmotic pressure at *z* is evaluated by integration of eq 1
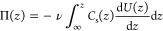
2where we have assumed Π(∞) → 0 as *C*_s_(∞) → 0 since the applied potential grows unbounded with distance from the origin. Given the salt concentration as a function of the position, we can determine the osmotic equation-of-state for the solute in solution, Π(*C*_s_).

From a practical point of view, the solute concentration profile would be evaluated via molecular simulation. In these simulations, the chemical potentials of both water and the solutes are expected to be uniform throughout the simulation box at equilibrium. While water’s chemical potential, μ_w_, arises from its interactions with other water molecules and the solutes, the solute’s chemical potential is
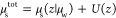
3where μ_s_(*z*|μ_w_) is the solute’s chemical potential arising from interactions of the solutes with water and the other solutes at constant μ_w_ and μ_s_^tot^ is the total solute chemical potential that includes the effect of the external potential. So while μ_s_^tot^ is independent of position within the simulation at equilibrium, μ_s_ is not. The osmotic pressure is subsequently determined by assuming that μ_s_(*z*|μ_w_) is equal to the solute’s chemical potential in a bulk solution at concentration *C*_s_(*z*). Specifically, at fixed temperature (d*T* = 0) and water chemical potential (dμ_w_ = 0), we obtain from the Gibbs–Duhem equation
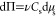
4where Π is the osmotic pressure, which is substituted for *P* when μ_w_ is constant. When the total solute chemical potential is fixed (dμ_s_^tot^ = 0), it follows from eq 3 that
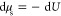
5

Substituting eq 5 into eq 4 yields
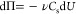
6

When the derivative is taken with respect to *z*, this expression reduces to eq 1, demonstrating that the osmotic pressure obtained from the force balance conforms to that obtained following a thermodynamic path. To be thermodynamically consistent, however, we expect the results obtained from the osmotic force balance to be independent of the applied external potential so that *C*_s_(*z*) is representative of the bulk solute concentration at a given osmotic pressure.

### Noninteracting Solutes

In the case of a noninteracting solute, the chemical potential is
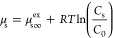
7where μ_s∞_^ex^ is the excess chemical potential of the solute at infinite dilution, *R* is the ideal gas constant, and *C*_0_ is a reference solute concentration. Substituting this expression into eq 5 and integrating, the solute concentration as a function of *z* is
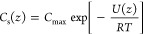
8where *C*_max_ is the maximum solute concentration at the external potential’s minimum. Substituting this equation into eq 2, the osmotic equation-of-state is found to be
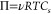
9

Thus, as expected, the osmotic pressure for noninteracting solutes is governed by the ideal gas law.

### Molar Description of Aqueous Electrolyte Solutions

The excess chemical potential of an aqueous electrolyte in solution at constant μ_w_ can be described by the inclusion of an activity coefficient^28,38,39^
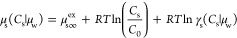
10awhere
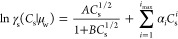
10b

The activity coefficient expression (eq 10b) is termed a modified Debye–Hückel equation (modDH). The first term on the right-hand side of eq 10b corresponds to the extended Debye–Hückel expression for the free energy of salts in solution. While *B* is assumed here to be a fitting parameter, the constant *A* is
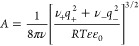
11where *q*_*j*_ indicates the charge on the cation (*j* = +) or anion (*j* = −), ε is the solvent’s dielectric constant, and ε_0_ is the permittivity of free space. The summation on the right-hand side of eq 10b is an empirical polynomial correction to the extended Debye–Hückel expression up to the power *i*_max_ with fitted constants α_*i*_. Integrating eq 5 from the potential minimum at the origin [where *U*(0) = 0] to *z* and substituting in eq 10a yields
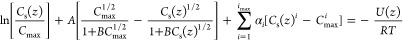
12

The *B* and α_*i*_ parameters in this expression can be fitted to the simulation results for *C*_s_(*z*). On the other hand, the maximum salt concentration, *C*_max_, is determined by ensuring the fit gives the correct number of ions in solution
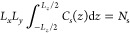
13where *L*_*j*_ denotes the length of the simulation box in the *j* (=*x*, *y*, or *z*) direction, whose coordinates run from −*L*_*j*_/2 to *L*_*j*_/2 with the origin at the center of the box. Inversion of eq 12 to find a simple analytical expression for *C*_s_(*z*) is not possible, in general. Nevertheless, eq 13 can be numerically integrated by substituting values of *C*_s_ less than *C*_max_ into eq 12 and determining the value of *z* that satisfies this expression. In the case of a harmonic potential
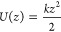
14where *k* is the spring constant, *z* is determined as
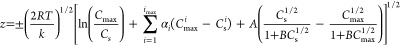
15

Once eq 12 has been fitted to the simulation results, the osmotic pressure is determined by substituting eq 10 into eq 4 and integrating
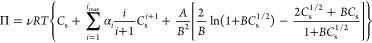
16

From the point of view of the force balance (eq 1), it is most natural to assume that the osmotic pressure is a function of *C*_s_. This is not necessarily the case following alternate thermodynamic approaches for describing osmotic equilibrium.

### Molal Description of Aqueous Electrolyte Solutions

Experimentally the chemical potential of water in solution is typically determined at ambient pressure using a range of techniques including, but not limited to, freezing point depression, vapor pressure, and dew point measurements.^39−41^ The osmotic pressure is then determined as the pressure increment required to raise water’s chemical potential in the mixture to that of pure water at atmospheric pressure. These approaches effectively fix the system at atmospheric pressure, which is distinct from the force balance. In this case, water’s chemical potential is determined from the solute’s chemical potential at fixed temperature (d*T* = 0) and pressure (d*P* = 0) using the associated Gibbs–Duhem relationship
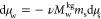
17where *m*_s_ (=*N*_s_/(*M*_w_^kg^*N*_w_)) is the solute molality and *M*_w_^kg^ is the molecular weight of water in kg/mol. More generally, *M*_*i*_^*j*^ is the molecular weight of solution component *i* in units of *j* = g or kg per mol. Thus, when pressure is fixed, the solute molality is the natural variable to describe the solution composition following eq 17. As such, the majority of experimental results for the activity of water in salt solutions and the osmotic pressure are reported as a function of molality.

The salt’s chemical potential in solution can be determined by adapting the modDH expression (eq 10) to a molal dependence at fixed pressure
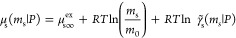
18awhere
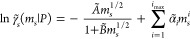
18b

Considering the relationship between the solute concentration and molality in the low concentration limit, *A* and *B* from eq 10 can be related to *Ã* and *B̃* as
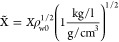
19where *X* is *A* or *B* and ρ_w0_ is the density of pure liquid water in g/cm^3^.^39^ As with eq 10, the parameters *B̃* and α*~*_*i*_ are fitting parameters.

For a constant pressure description of electrolyte solutions, the osmotic pressure is determined as the pressure required to raise the free energy so that water’s activity is 1, so that the chemical potential of water in the mixture at pressure *P* + Π is equal to that of pure water at pressure *P*. The activity of water at fixed pressure, *a*_w_(*m*_s_|*P*), is determined by substitution of eq 18 into eq 17 and integration along a line of constant pressure.
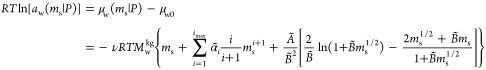
20where μ_w0_ is the chemical potential of pure water at pressure *P*. Assuming that the solution is incompressible, the osmotic pressure is determined as
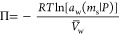
21where *V̅*_w_ is the partial molar volume of water in the mixture, which can be estimated from the solution density as described below. Alternately, water’s partial molar volume in salt solutions does not vary significantly from that of pure water, less than ∼3% difference over the salt concentration ranges considered here. The osmotic pressure is then well approximated using the molar volume of pure water in the absence of any information about water’s volumetric properties in mixtures.

### Solution Densities

An additional thermodynamic variable that can be derived from osmotic force balance simulations is the dependence of the density on the solution composition. Specifically, the water concentration as a function of position, *C*_w_(*z*), is obtained from these simulations along with the solute concentration. The solution density profile within the simulation box can subsequently be evaluated, albeit at a constant μ_w_. The density at a constant *P* can then be determined from knowledge of the osmotic pressure and an estimate for the solution compressibility. While the solution concentration changes with the pressure, its molality does not. Here, then, we assume that the density at constant μ_w_ or *P* is described by the expression
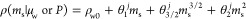
22where ρ_w0_, the density of pure water, and the coefficients θ_*i*_^*j*^ are fitting parameters determined along lines of constant pressure (*j* = *P*) or water chemical potential (*j* = μ, where the subscript *w* has been dropped for simplicity). The solution density at constant *P* can be approximated as
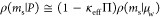
23where κ_eff_ is the effective compressibility of the solution. We estimate the compressibility of the salt solutions in the Results and Discussion. The partial molar volume of water as a function of the salt molality can be estimated from eq 22 as
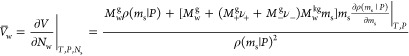
24which we use to evaluate the water’s activity from the osmotic pressure using eq 21.

### Fitting the Alternate the Osmotic Pressure

Here, we describe our procedure for fitting constant μ_w_ or *P* descriptions of the osmotic pressure to our simulation results. In this fitting, *A* is determined from Debye–Hückel theory (eq 11), while the *A* and *Ã* terms and *B* and *B̃* terms are assumed to be related to one another through eq 19. This approximation is reasonable given that the terms appear in the extended Debye–Hückel contribution for the electrostatic free energy best apply at a low concentration where the difference between the constant μ_w_ and *P* is minimal. An initial value of *B* = 1.5 M^–1/2^ was used for each fitting and then varied to minimize the difference between the osmotic pressures evaluated following eqs 16 and 21. The α_*i*_ terms appearing in eq 10b are determined by a least-squares fit of eq 12 to the salt concentration profile determined from simulation under the mass balance constraint (eq 13). The α̃_*i*_ terms appearing in eq 18b are then determined by a least-squares fit of eq 21 to the osmotic pressures evaluated from eq 2 by numerical integration of the simulation concentration profile. We found that a value of *i*_max_ = 2 provided an excellent description of our simulation results over the simulated range of salt concentrations, up to ∼3.5 M (corresponding to ∼4 molal).

## Molecular Simulation Methods

Osmotic force balance molecular dynamics simulations of aqueous electrolyte solutions were conducted in the canonical ensemble^42^ using GROMACS 2020.7.^43^ We considered salts composed of all combination of the alkali metal cations Li^+^, Na^+^, K^+^, Rb^+^, and Cs^+^ with the halide anions Cl^–^, Br^–^, and I^–^, for a total of 15 monovalent 1:1 electrolytes in water (LiCl, LiBr, LiI, NaCl, NaBr, NaI, KCl, KBr, KI, CsCl, CsBr, CsI, RbCl, RbBr, and RbI). The fluoride anion proved to be problematic as a result of solubility issues that arose during the simulations, resulting in the fluoride salts being neglected. The temperature was set to 25 °C and was controlled using the Nosé–Hoover thermostat.^44,45^ Water was modeled using the TIP4*P*/2005 potential.^46^ The dielectric constant of the model was taken to be 59.1 for fitting to the free energy models above.^47^ The ions were modeled using the potential parameters reported by Joung and Cheatham^48^ parametrized for TIP4P/Ew water,^49^ which have also been shown to accurately capture the single-ion properties of the monovalent salts in TIP4*P*/2005 water.^50^ Cross interactions of the ions with water were evaluated using Lorentz–Berthelot combining rules (Table 1).^42^ While initial cross interactions between the cations and anions were evaluated using Lorentz–Berthelot combining rules,^42^ those interactions were subsequently optimized to reproduce the experimental osmotic pressures as described below. Lennard-Jones interactions were evaluated out to 9 Å with a long-range mean field correction for potential truncation effects. Electrostatic interactions were evaluated using Particle Mesh Ewald Summation.^51^ The internal holonomic constraints for water were fixed using the LINCS algorithm.^52^ The equations-of-motion were integrated with a 2 fs time step. Simulations were equilibrated for 25 ns, followed by a production run of 200 ns to determine equilibrium averages.

**Table 1:** Cation–Anion Lennard-Jones Cross-Interaction Parameters for the Joung–Chetham^48^ Potential Determined by Lorentz–Berthelot Combining Rules and Optimized to Reproduce the Experimental Osmotic Pressure Following eq 25^a^

	Lorentz–Berthelot			optimized	
salt	σ_+–_	ε_+–_	χ	σ_+–_	ε_+–_
LiCl	3.1787	17.524	0.75	3.4895	10.014
LiBr	3.1859	28.283	0.90	3.5456	14.886
LiI	3.3498	33.141	1.06	3.7786	16.088
NaCl	3.5511	22.303	–0.32	3.3300	32.798
NaBr	3.5582	35.996	–0.41	3.2587	61.010
NaI	3.7222	42.178	–0.55	3.2584	93.729
KCl	3.8754	28.728	0.25	4.0223	22.982
KBr	3.8825	46.366	–0.30	3.6585	66.237
KI	4.0465	54.329	0.20	4.1713	45.274
RbCl	3.9814	35.765	0.50	4.2598	23.843
RbBr	3.9886	57.723	0.43	4.2336	40.366
RbI	4.1525	67.637	0.35	4.3655	50.102
CsCl	4.1409	34.129	0.34	4.3479	25.469
CsBr	4.1480	55.083	0.22	4.2878	45.150
CsI	4.3119	64.544	0.00	4.3119	64.544

a The optimal value of χ is also reported. Notably for CsI, the unmodified potential (χ = 0) produced the optimal result. The units for the Lennard-Jones diameter and well-depth are Å and K, respectively.

**Figure 1:**
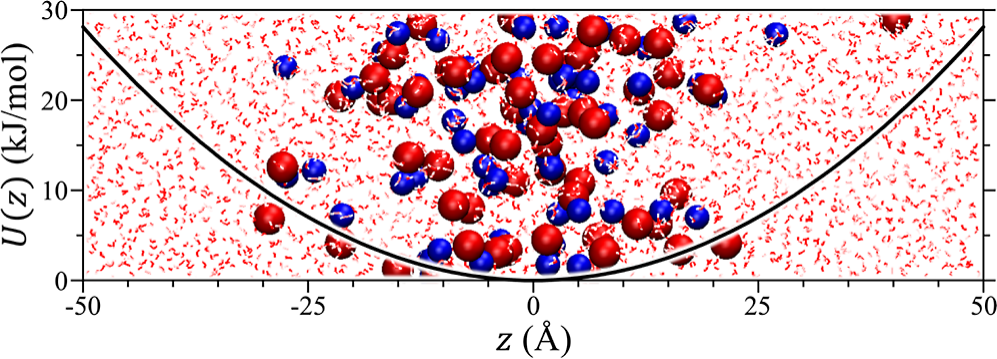
Harmonic potential, φ, applied to the Na^+^ and Br^–^ ions in aqueous solution in a 30 × 30 × 100 Å cell overlaid on top of the final snapshot from the simulation. The Na^+^ and Br^–^ ions as shown as the blue and red van der Waals spheres, respectively, while water is illustrated by the background stick representation. The simulation snapshot was visualized using VMD.^64^

The primary simulation box used was a rectangular box of 30 × 30 × 100 Å along the *x*, *y*, and *z* directions. The simulations included 60 cations and 60 anions (*N*_s_ = 60) and approximately 3000 waters, except in the case of CsI where *N*_s_ = 40 to ensure the maximum concentration fell below the solubility limit. A harmonic potential (eq 14) was applied to the ions to confine the ions near the center of the *z*-axis while forming a reservoir of pure water on either end of the simulation box along *z*. The spring constant *k* was adjusted so that the salt concentration at the *z* origin was ∼3.5 M. An equilibrated snapshot from a simulation of NaBr in water is shown in Figure 1 illustrating the impact of the harmonic restraint on the distribution of cations and anions in the simulation. Since the simulations were conducted in the canonical ensemble, to ensure the chemical potential of water was effectively equal to that of pure water at 25 °C and atmospheric pressure, the number of waters in the final simulation for each ion pair was adjusted in a series of shorter preliminary simulations so that the density of water at either end of the box was 0.997 ± 0.0005 g/cm^3^, the density of pure TIP4*P*/2005 water at ambient conditions (the resulting bulk pressures lie within ±10 atm of atmospheric pressure). The density of pure water, ρ_w0_, was determined by fitting eq 22 to our results. In addition to simulations with a 30 × 30 × 100 Å simulation box, we also simulated NaBr and RbCl in boxes with dimensions of 30 × 30 × 50 Å and 28.23 × 28.23 × 28.23 Å to perform thermodynamic consistency checks. The spring constants and specific numbers of waters and ions used in each osmotic force balance simulation are reported in Table S1 in the Supporting Information.

Following Milner,^23^ we fit the simulation results for the osmotic pressure to the experimental equations-of-state by modifying the repulsive portion of the cross interaction between unlike ions. This is achieved multiplying the repulsive *r*^–12^ (*r* represents the distance between ions) portion of the cross interaction by 1 + χ, where χ (>−1) is an adjustable optimization parameter, while not modifying the attractive *r*^–6^ portion of the potential
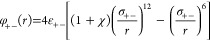
25

The effect of this fitting scheme is to modify the Lennard-Jones well-depth and diameter as
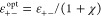
26aand
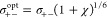
26b

The best value of χ was determined by minimizing the mean square difference between the experimental and simulation results for the osmotic pressure as a function of the salt concentration. Experimental results for the osmotic pressures of the monovalent salts were taken from the extensive tables compiled by Hamer and Wu.^39^ Initial simulations are conducted with the cross interaction well-depth and diameter obtained from Lorentz–Berthelot combining rules (χ = 0). A series of additional simulations with differing values of χ were then run to find the optimal interaction. We typically started with large variations in χ to bound the optimal result, followed by additional simulations to zero in on a best fit. This would typically take ∼4 additional simulations to find a reasonable estimate of the optimum value following our initial simulation with χ = 0.

To compare the salt solution densities we obtained from the osmotic force balance simulations against those at atmospheric pressure, we conducted simulations of bulk 1, 2, and 3 molal aqueous solutions of LiI, KCl, and CsBr in the isothermal–isobaric ensemble. The pressure was maintained with a Parinello–Raman barostat.^53^ The ions were modeled by using the optimized potentials from the force balance simulations. The numbers of waters and ions in each of these simulations is reported in the Supporting Information (Table S2). These simulations were equilibrated for 25 ns, followed by a 200 ns run to evaluate the thermodynamic averages.

## Results and Discussion

### Thermodynamic Consistency

A necessary condition for the application of the osmotic force balance to accurately capture the thermodynamic properties of aqueous electrolyte solutions is that the results derived are independent of the specifics of the simulation boxes used. One potential consistency check is to fit the chemical potentials from one simulation of a given electrolyte and predict the concentration profile in a different simulation box by using the fitted chemical potential. Milner^23^ proposed an alternate check that avoids having to fit the chemical potentials by comparing the concentration profiles from two simulations (1 and 2) with different sizes where the concentrations of water and salt in the two simulations are equal
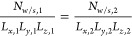
27while the harmonic constants of the external potential in the two simulations are related to one another as
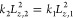
28

When the concentration profiles in the two simulations are plotted as a function of the dimensionally reduced *z*-coordinate, 2*z*/*L*_*z*_, they should collapse on top of one another if they are consistent.

**Figure 2:**
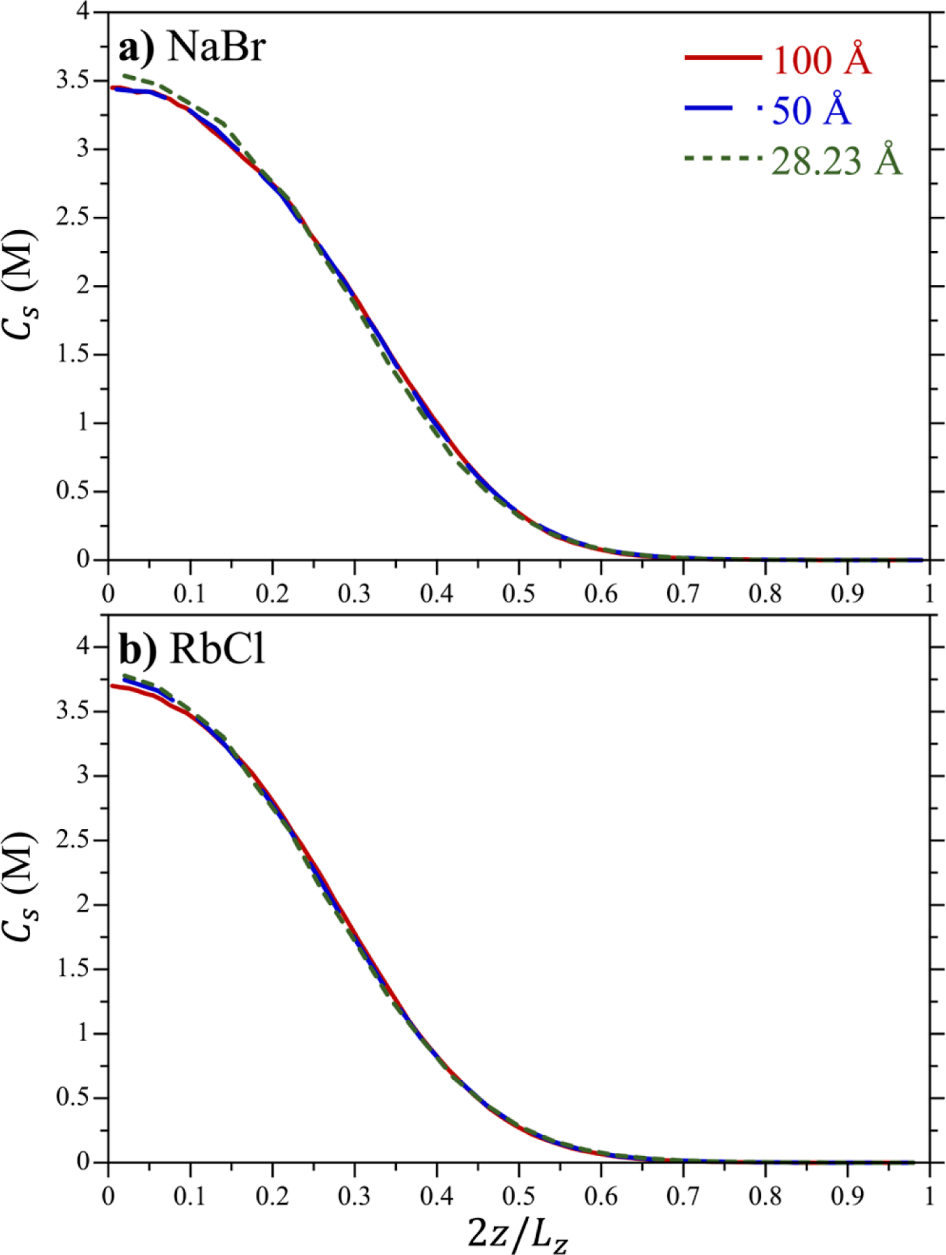
Aqueous electrolyte concentration, *C*_s_, profiles as a function their reduced *z* position, 2*z*/*L*_*z*_, for boxes of varying box sizes and harmonic restraint constants, *k*. The figures illustrate results for (a) NaBr and (b) RbCl with χ = 0. The concentration profiles have been averaged about the line of symmetry (*z*. = 0) to improve statistical accuracy. Results are shown for boxes with *L*_*z*_ = 100 Å (box dimensions of 30 × 30 × 100 Å, with *k* = 0.025 kJ/(mol Å^2^) for NaBr and 0.0225 kJ/(mol Å^2^) for RbCl), 50 Å (30 × 30 × 50 Å, with *k* = 0.1 kJ/(mol Å^2^) for NaBr and 0.09 kJ/(mol Å^2^) for RbCl), and 28.23 Å (28.23 × 28.23 × 28.23 Å, with *k* = 0.3137 kJ/(mol Å^2^) for NaBr and 0.2823 kJ/(mol Å^2^) for RbCl). The simulation lines are defined in the legend of (a).

To demonstrate thermodynamic consistency of the osmotic force balance, the concentration profiles for two representative salts, NaBr and RbCl, for three different simulations spanning a factor of 4 in volume are reported in Figure 2. These profiles exhibit a maximum at the center of the box (*z* = 0 Å) that tails off with increasing distance from the minimum in the applied potential. While at first glance these distributions may appear to be Gaussian, they are not normally distributed as a result of intermolecular interactions, as demonstrated below. More importantly, the concentration profiles of the different simulations collapse onto one another for each salt, indicative of thermodynamic consistency. The simulation of NaBr with *L*_*z*_ = 28.23 Å (Figure 2a) exhibits the most significant differences between the concentration profiles, although the difference is small. This is the smallest simulation cell considered; however, we attribute this discrepancy to the fact that consistency is expected to breakdown as the external potential forces get larger as the box gets smaller. Nevertheless, the differences of this simulation with those performed using larger boxes are small, giving us confidence that we can obtain reliable results for the chemical potential as long as we use large enough simulation cells. Resultantly, we fit the chemical potentials below to simulations performed using the largest box considered, *L*_*z*_ = 100 Å.

**Figure 3:**
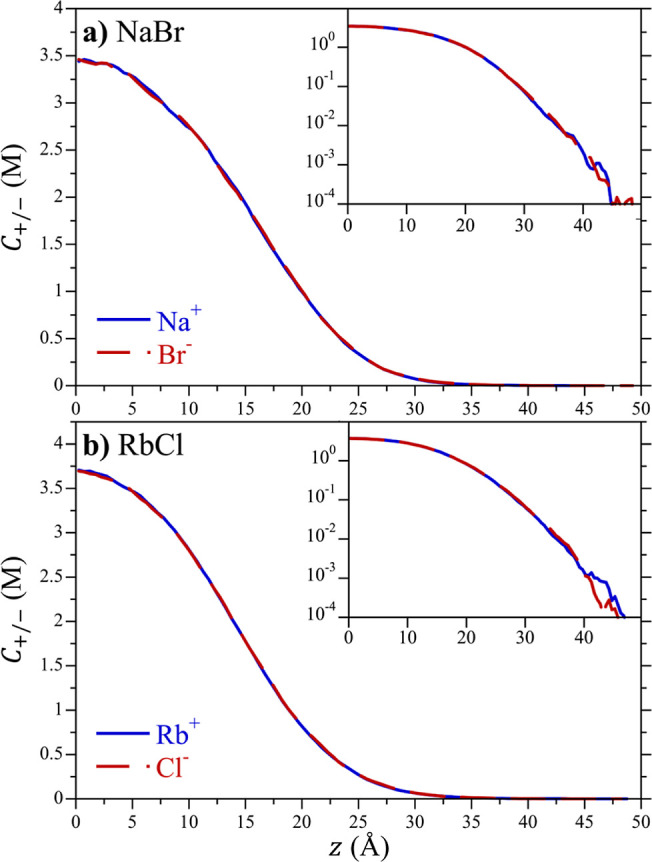
Cation and anion concentration as a function of *z* for (a) NaBr and (b) RbCl in a simulation box with dimensions 30 × 30 × 100 Å with χ = 0. The concentration profiles have been averaged about the line of symmetry (*z* = 0) to improve statistical accuracy. The inset figures show the concentration on a logarithmic scale to more clearly see the ion concentrations at down to sub mM concentrations. The simulation lines are defined in the legend of each figure.

A second consistency condition for the application of the osmotic force balance is that the simulations are locally electroneutral. Specifically, the charge densities of cations, *q*_+_*C*_+_, and anions, *q*_–_*C*_–_, should cancel each another as a function of *z* (i.e., *q*_+_*C*_+_(*z*) + *q*_–_*C*_–_(*z*) = 0). Milner^23^ demonstrated that the bulk concentrations of cations and anions can differ when the osmotic pressure is determined by the forces acting on a membrane-like restraint wall that induces ion structuring at its interface. It can be difficult in this case to even ascribe a specific salt concentration to the observed pressure, complicating the determination of the osmotic equation-of-state. Milner, however, did not demonstrate local electroneutrality for the osmotic force balance technique itself. The concentration profiles of the cations and anions comprising the NaBr and RbCl systems with *L*_*z*_ = 100 Å are reported in Figure 3. The anion and cation concentration profiles of both salts match one another over the entire box length, which is expected for monovalent salts if they are locally electroneutral. This holds even down to submolar concentrations (Figure 3 inset). Indeed, statistical differences between the anion and cation concentrations are observed only below 1 mM (0.001 M) in these simulations, which can be attributed to deficiencies in sampling such low concentrations. Indeed, 1 mM corresponds to one ion pair per 55,000 waters, which is well above the number of waters in our simulations (∼3000 waters). Similarly good results are found for simulations of the other ions in water (not reported here). When fitting the modDH chemical potential expression (eq 10) to our simulation concentration profiles below, we only consider data for concentrations above 10 mM, which is an order of magnitude above the concentration for which electroneutrality fails as a result of poor sampling. Taken together, the demonstrated box size independence and electroneutrality give us confidence that thermodynamically consistent results for the osmotic pressure and chemical potential of ions in water can be obtained from osmotic force balance simulations.

### Osmotic Pressure Evaluation

**Figure 4:**
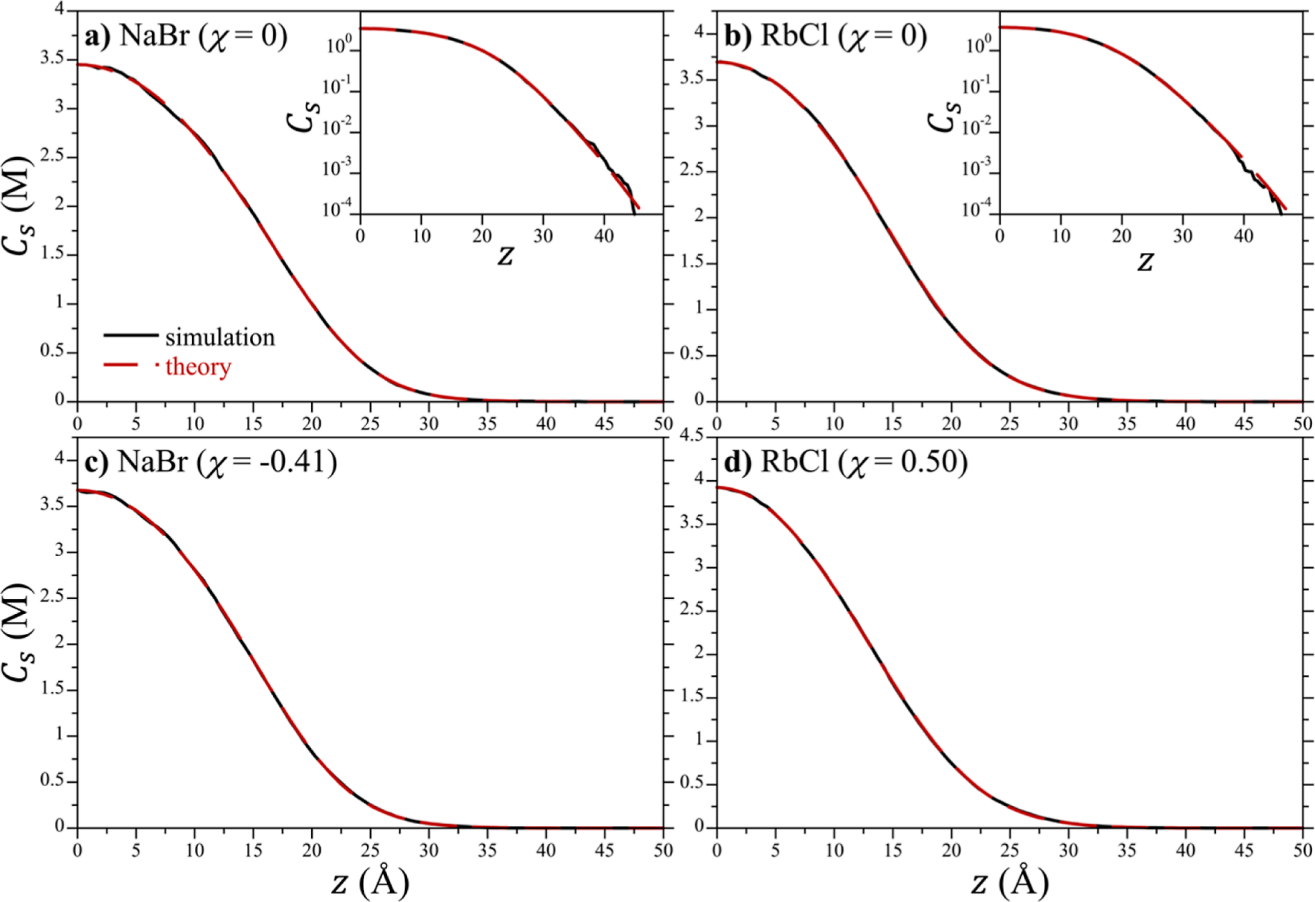
Comparison of the simulation concentration profiles of NaBr and RbCl against the fits of the modDH theoretical expression (eq 10a). (a,c) Results for NaBr using the original (χ = 0) and optimized (χ = −0.41) Lennard-Jones cross interactions, respectively. (b,d) Results for RbCl using the original (χ = 0) and optimized (χ = 0.50) Lennard-Jones cross interactions, respectively. The concentration profiles have been averaged about the line of symmetry (*z* = 0) to improve statistical accuracy. The inset figures in (a,b) show the concentration on a logarithmic scale to more clearly see the ion concentrations at down to sub mM concentrations. The figure symbols are defined in the legend of (a).

The concentration profiles for NaBr and RbCl using the unmodified Lennard-Jones potentials (χ = 0) determined from simulation are compared to the fits of the modDH equation for the chemical potential in Figure 4a,b, respectively. These concentration profiles are described with quantitative accuracy by the modDH equation down into the sub-Molar concentration regime (Figure 4a,b inset). Similarly good agreement is found for all the other simulated salts as reported in the Supporting Information (Figures S1–S13). The fit parameters of the modDH equation (eq 10) to the concentration profiles for the salts with χ = 0 are reported in the Supporting Information (Table S3).

**Figure 5:**
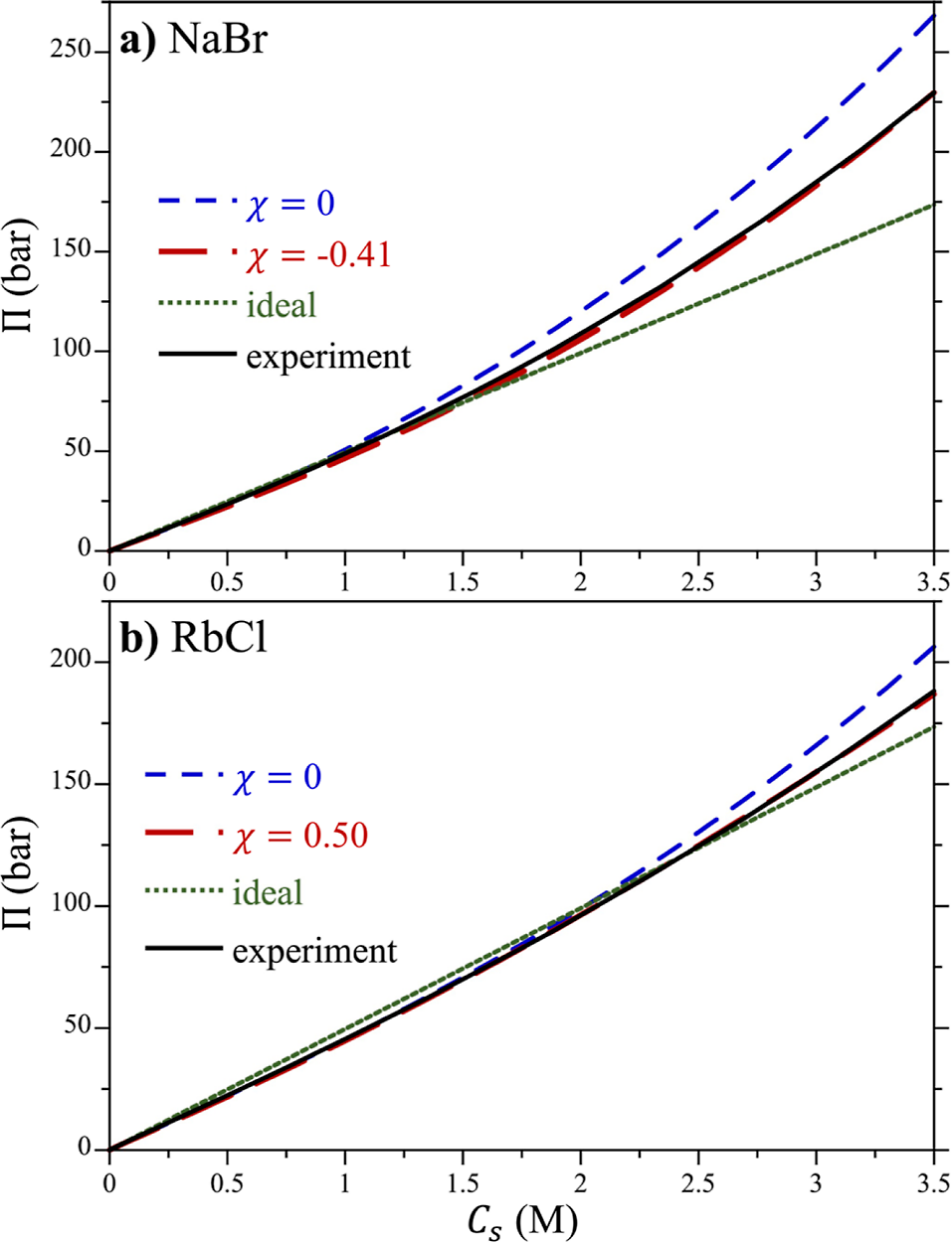
Osmotic pressure of (a) NaBr and (b) RbCl in water as a function of the salt concentration. Results obtained from simulation using original (χ = 0) and optimized (χ = −0.41 and 0.50 for NaBr and RbCl, respectively) are compared against experiment and the ideal gas law. The experimental results are taken from Hamer and Wu.^39^ The figure symbols are defined in the legend of each figure.

The osmotic pressures associated with the fits of the concentration profiles for NaBr and RbCl with χ = 0 are reported in Figure 5, while results for all other simulated salts are reported in the Supporting Information (Figures S14–S26). At low salt concentrations, both salts effectively behave ideally, although slight negative deviations are observed as a result of screening, as described by the Debye–Hückel limiting law. The osmotic pressures determined for both NaBr and RbCl using the unmodified Lennard-Jones potentials are greater than those in the corresponding experiments at higher concentrations. Nevertheless, both the simulation and experimental pressures are greater than would be obtained assuming the salts behaved like an ideal gas at an elevated concentration (*C*_s_ ≳ 2 M), resulting from multibody interactions of the salts in solution. Interestingly, NaBr exhibits a markedly greater pressure at elevated concentrations than RbCl from both simulation and experiment, indicative of distinct differences between how these salts behave in solution.

To bring the osmotic pressures from simulation into agreement with experiment, the cross interactions between the anions and cations were modified following eq 25. As reported in Figure 5, the simulated osmotic pressures for NaBr and RbCl can be brought into excellent agreement with experiments following this approach. Similarly good agreement is obtained for all of the other simulated salts (Figures S14–S26 in the Supporting Information). The optimized Lennard-Jones cross-interaction parameters obtained following this scheme for all the simulated salts are reported in Table 1. (The unmodified interactions for CsI were notably found to be optimal.) As with the original Lennard-Jones interactions, the modDH expression provides an excellent quantitative description of the concentration profiles of NaBr and RbCl obtained by using the optimized Lennard-Jones interactions. (Figure 4c,d. Results for the other simulated salts reported in Figures S1–S13 in the Supporting Information.) The fitted parameters of the concentration-dependent modDH expression for the chemical potential to the simulation results for all of the salts using the optimized Lennard-Jones interactions are reported in Table 2. Interestingly, while the predictions for osmotic pressures above using the unmodified Lennard-Jones cross interactions are too repulsive, the optimized values of χ for NaBr (χ = −0.41) and RbCl (χ = 0.50) differ in sign. Intuitively, for an overly repulsive pressure, we might expect the optimized χ would be negative to reduce the repulsion between the anions and cations, in line with what is observed for NaBr. RbCl, however, breaks this expectation. The osmotic pressure is the result of not only the cross interaction of anions with cations, however, but also from the interactions of the anions and cations with themselves and with water. We suspect that to achieve agreement between simulation and experiment at higher concentrations, the change in the cross interactions plays a nontrivial secondary role in the interactions of the anions and cations with their similarly charged brethren. Moreover, our simulations neglect any effects of polarization on intermolecular interactions, which could lead to additional sources of error in the simulations. Here, we have optimized the interactions to obtain agreement with experiment, but not the molecular origin of this behavior, which would require deeper investigation. Indeed, a range of negative and positive results for χ were found for all of the salts with no discernible correlation between the anion and cation pairs, suggesting potential new avenues of investigation.

**Table 2:** Parameters of the Modified Debye–Hückel Expressions for the Free Energy of the Simulated Salts in Aqueous Solution; Table (a**)** Reports Fits Determined Directly from the Osmotic Force Balance Simulations at Constant μ_w_; The Units of *A*, *B*, α_1_, and α_2_ Are M^–1/2^, M^–1/2^, M^–1^, and M^–2^, Respectively; Table (b**)** Reports Fits at Constant *P* to the Osmotic Pressures; The Units of *Ã*, *B̃*, α*~*_1_, and α*~*_2_ Are molal^–1/2^, molal^–1/2^, molal^–1^, and molal^–2^, Respectively

(a) Coefficients for μ_s_(*C*_s_|μ_w_) along a Line of Constant μ_w_ (eq 10 with A = 1.7964)								
salt	LiCl	LiBr	LiI	NaCl	NaBr	NaI	KCl	KBr
*B*	1.4374	1.6147	3.0262	1.5111	1.3145	2.0662	1.0587	0.98099
α_1_	0.23869	0.25376	0.19784	9.78620 × 10^–^^2^	0.17100	0.14694	0.17598	0.21795
α_2_	2.4656 × 10^–^^2^	4.4561 × 10^–^^2^	8.5002 × 10^–^^2^	2.0657 × 10^–^^2^	2.2058 × 10^–^^2^	4.3132 × 10^–^^2^	–3.7167 × 10^–^^3^	–4.3250 × 10^–^^4^

salt	KI	RbCl	RbBr	RbI	CsCl	CsBr	CsI	
*B*	1.9396	1.2829	1.2525	1.7599	1.0988	1.3442	1.5623	
α_1_	0.27765	0.14017	0.18510	0.17828	0.10968	0.12351	0.10900	
α_2_	–2.5242 × 10^–^^2^	–1.0095 × 10^–^^3^	–9.9174 × 10^–^^3^	–1.5846 × 10^–^^2^	5.5867 × 10^–^^3^	7.8015 × 10^–^^4^	–1.8300 × 10^–^^3^	

(b) Coefficients for μ_s_(*m*_s_|*P*) along a Line of Constant *P* (eq 18 with *Ã* = 1.7937)								
salt	LiCl	LiBr	LiI	NaCl	NaBr	NaI	KCl	KBr
*B̃*	1.4352	1.6123	3.0217	1.5088	1.3125	2.0631	1.0571	0.97951
α*~*_1_	0.19841	0.21075	0.16346	0.10009	0.16772	0.15070	0.12085	0.17804
α*~*_2_	4.4793 × 10^–^^3^	7.6354 × 10^–^^3^	1.4761 × 10^–^^2^	1.1422 × 10^–^^2^	9.1095 × 10^–^^3^	2.0240 × 10^–^^2^	–9.9936 × 10^–^^3^	–1.0053 × 10^–^^2^

salt	KI	RbCl	RbBr	RbI	CsCl	CsBr	CsI	
*B̃*	1.9366	1.2809	1.2506	1.7573	1.0971	1.3422	1.5599	
α*~*_1_	0.12757	6.5727 × 10^–^^2^	7.7266 × 10^–^^2^	2.2019 × 10^–^^2^	4.2305 × 10^–^^2^	3.1092 × 10^–^^2^	3.5879 × 10^–^^2^	
α*~*_2_	–1.9203 × 10^–^^2^	–7.0482 × 10^–^^3^	–1.0671 × 10^–^^2^	–8.7860 × 10^–^^3^	–3.6882 × 10^–^^3^	–5.1292 × 10^–^^3^	–5.4064 × 10^–^^3^	

### Electrolyte Solution Thermodynamics

**Figure 6:**
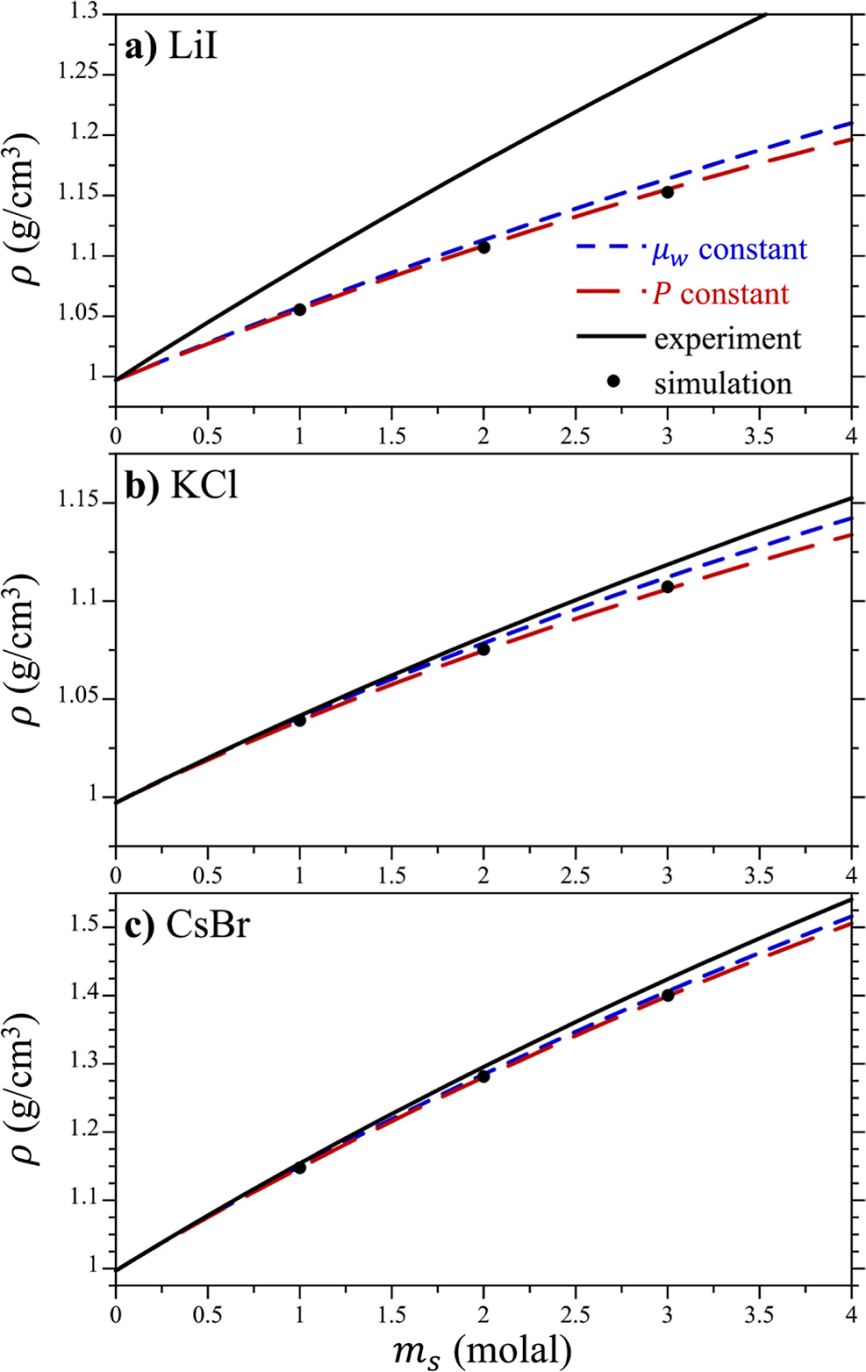
Density of the aqueous salt solutions as a function of the solution molality. Figures report results for (a) LiI, (b) KCl, and (c) CsBr. We compare results from the osmotic force balance simulations (μ_w_ constant), osmotic force balance simulations corrected to 1 bar pressure (*P* constant), experiment (experiment), and bulk simulations at 1 bar pressure (simulation). The lines were fit to eq 22. The figure symbols are defined in the legend of (a).

Before the free energies of the salts at atmospheric pressure can be determined, we must determine the volumetric properties of the solution to evaluate the activity of water from the osmotic pressure (eq 21). This, in turn, requires an estimate of the compressibility of the salt solutions (eq 23). The aqueous densities of LiI, KCl, and CsBr determined from the osmotic force simulations using the optimized Lennard-Jones interactions, ρ(*m*_s_|μ_w_), are compared against experiment in Figure 6. The reported simulation densities are lower than those of their experimental counterparts in this figure. Perhaps, this is not surprising given that the unmodified potentials were assessed by their ability to reproduce single ion properties, but not other solution properties.^50^ Indeed, the nonpolarizable Madrid model for aqueous electrolytes required optimization of not only the cross anion–cation Lennard-Jones interaction but also the anion–water oxygen and cation–water oxygen interactions and scaled-charges as well to reproduce a wide-range of solution properties.^26,54^ That said, the predicted solution densities for KCl (Figure 6b) and CsBr (Figure 6c) only slightly underpredict the experimental densities, although the predictions for LiI (Figure 6a) are significantly off. Notably, classical simulations of Li^+^ ions have reported a range of first-shell coordination numbers in water, indicative of uncertainty in the models.^55^ Given that the experiments were conducted at atmospheric pressure, while the simulations are conducted under osmotic stress, we should expect some error. Removing the effect of the osmotic pressure on the solution densities, however, would only reduce the predicted densities and increase the difference between simulation and experiment.

A more appropriate comparison to assess the accuracy of the predicted densities is to compare the results from the force balance simulations against bulk salt solution simulations performed at ambient pressure. To this end, atmospheric pressure simulation results for LiI, KCl, and CsBr solutions at concentrations of 1, 2, and 3 molal using the optimized Lennard-Jones cross interactions are reported in Figure 6. While qualitatively similar to the results obtained at constant μ_w_, the densities at atmospheric pressure are slightly lower, with the difference between the two simulation series increasing with increasing concentration. This difference reflects the growing compression of the solution as a result of the osmotic pressure with an increasing concentration. While the solution compressibility depends on the concentration and the specific salts in solution, it is not expected to differ significantly from that of water. We subsequently assume we can use an effective value of the compressibility, κ_eff_, across all salts and solution concentrations to obtain an estimate of the solution density at atmospheric pressure. Excellent agreement is observed between the predictions for ρ(*m*_s_|*P*) using eq 23 and the bulk atmospheric pressure simulations using a value of κ_eff_ = 3.95 × 10^–5^ bar^–1^. Interestingly, κ_eff_ is only 20% lower than κ_w_ for TIP4*P*/2005 water (=4.65 × 10^–5^ bar^–1^). In the absence of information about the compressibility of these solutions, reasonable results can be obtained assuming κ_eff_ = κ_w_. Fits of the densities at constant μ_w_ and *P* to eq 22 for all of the simulated salts using the optimized Lennard-Jones potentials are reported in Table 3. Results for simulations using the unmodified Lennard-Jones interactions (χ = 0) are reported in the Supporting Information (Table S4).

**Table 3:** Fits of eq 22 to the Simulation Densities of Aqueous Salt Solutions as a Function of the Salt Molality along Lines of Constant μ_w_ and *P* at 25°C^a^

salt	LiCl	LiBr	LiI	NaCl	NaBr	NaI	KCl	KBr
ρ_w0_	0.99702	0.99712	0.99713	0.99700	0.99695	0.99700	0.99713	0.99680
θ1^μ^	1.2346 × 10^–^^2^	3.9357 × 10^–^^2^	6.3490 × 10^–^^2^	6.2948 × 10^–^^2^	9.9941 × 10^–^^2^	0.13606	4.9510 × 10^–^^2^	9.9842 × 10^–^^2^
θ_3/2_^μ^	–2.3566 × 10^–^^3^	1.0385 × 10^–^^3^	–7.1690 × 10^–^^4^	–7.1889 × 10^–^^3^	–5.4726 × 10^–^^3^	–1.4879 × 10^–^^3^	–5.3635 × 10^–^^3^	–5.3608 × 10^–^^3^
θ_2_^μ^	2.7688 × 10^–^^4^	–1.6204 × 10^–^^3^	–2.2102 × 10^–^^3^	–4.1790 × 10^–^^5^	–1.0888 × 10^–^^3^	–3.1384 × 10^–^^3^	–6.2850 × 10^–^^4^	–1.8468 × 10^–^^3^
θ_1_^P^	1.0546 × 10^–^^2^	3.7330 × 10^–^^2^	6.1383 × 10^–^^2^	6.0936 × 10^–^^2^	9.7856 × 10^–^^2^	0.13343	4.7929 × 10^–^^2^	9.8248 × 10^–^^2^
θ_3/2_^P^	–2.0884 × 10^–^^3^	1.6557 × 10^–^^3^	–1.2910 × 10^–^^5^	–6.5261 × 10^–^^3^	–4.5989 × 10^–^^3^	2.3562 × 10^–^^4^	–5.4872 × 10^–^^3^	–5.3708 × 10^–^^3^
θ_2_^P^	–7.0290 × 10^–^^5^	–2.1963 × 10^–^^3^	–2.8867 × 10^–^^3^	–5.7990 × 10^–^^4^	–1.8555 × 10^–^^3^	–4.3985 × 10^–^^3^	–7.0140 × 10^–^^4^	–2.1235 × 10^–^^3^

salt	KI	RbCl	RbBr	RbI	CsCl	CsBr	CsI
ρ_w0_	0.99698	0.99676	0.99716	0.99686	0.99736	0.99734	0.99679
θ_1_^μ^	0.11226	8.5821 × 10^–^^2^	0.12109	0.15398	0.12948	0.16846	0.22188
θ_3/2_^μ^	–6.1732 × 10^–^^3^	–7.0817 × 10^–^^3^	–9.4249 × 10^–^^3^	–1.4705 × 10^–^^2^	–8.5014 × 10^–^^3^	–1.3048 × 10^–^^2^	–6.8022 × 10^–^^3^
θ_2_^μ^	–2.8402 × 10^–^^3^	–1.5060 × 10^–^^3^	–2.2284 × 10^–^^3^	–2.3955 × 10^–^^3^	–2.8228 × 10^–^^3^	–3.1837 × 10^–^^3^	–5.4566 × 10^–^^3^
θ_1_^P^	0.11113	8.4221 × 10^–^^2^	0.11975	0.15281	0.12785	0.16700	0.22035
θ_3/2_^P^	–7.3155 × 10^–^^3^	–7.2535 × 10^–^^3^	–1.0040 × 10^–^^2^	–1.5714 × 10^–^^2^	–8.5480 × 10^–^^3^	–1.3468 × 10^–^^2^	–7.1660 × 10^–^^3^
θ_2_^P^	–2.6182 × 10^–^^3^	–1.5783 × 10^–^^3^	–2.1754 × 10–^3^	–2.1964 × 10^–^^3^	–2.9858 × 10^–^^3^	–3.2468 × 10^–^^3^	–5.6528 × 10^–^^3^

a The units of ρ_w0_, θ_1_^*j*^, θ_3/2_^*j*^, and θ_2_^*j*^ are g/cm^3^, g/(cm^3^ molal), g/(cm^3^ molal^3/2^), and g/(cm^3^ molal^2^), respectively.

**Figure 7:**
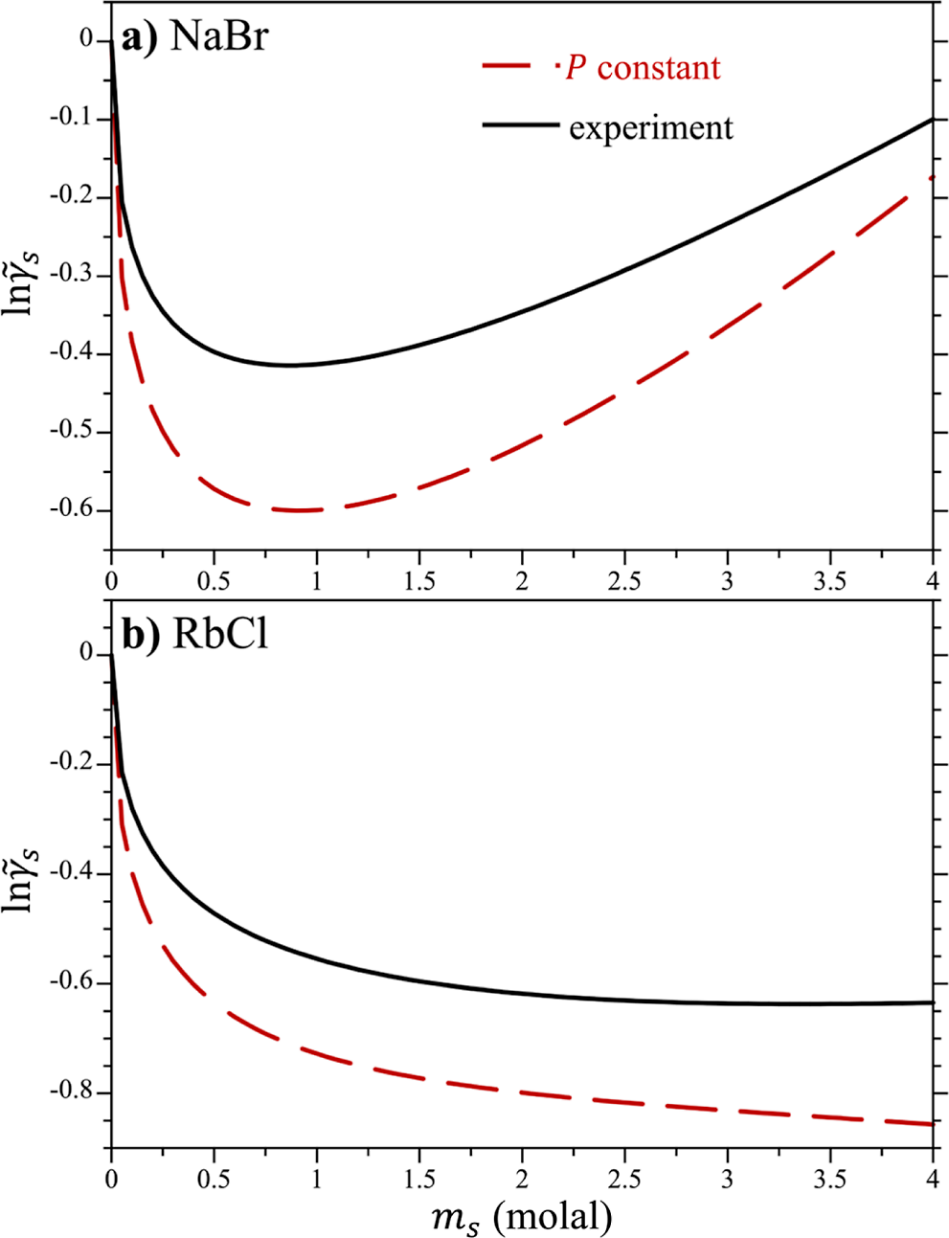
Activity coefficients of (a) NaBr and (b) RbCl in water at ambient pressure by fitting eq 20 to the osmotic pressures of the salts at 25 °C. The simulations were conducted using the optimized Lennard-Jones cross interactions. The experimental results are taken from Hamer and Wu.^39^ The figure symbols are defined in the legend of (a).

Since the fits of the constant *P* expression of the modDH theory (eq 18) were performed to reproduce the simulation osmotic pressures, which themselves had been fitted to the experiment, the theory is expected to accurately capture the activity of water at ambient conditions given its simple proportionality with the osmotic pressure (results not shown). It is not assured, however, that the activity coefficients of the salts in solution will match experiment. The constant *P* activity coefficient fits of NaBr and RbCl are subsequently reported in Figure 7 to assess the accuracy of the simulations in capturing the experimental values. Activity coefficients for all the other simulated salts are reported in Figures S27–S39 in the Supporting Information. For NaBr and RbCl, the simulation results are in qualitative agreement with experiment. In the case of NaBr (Figure 7a), both the simulation and experimental results exhibit an initial steep drop, followed by a minimum in the neighborhood of a 0.5 to 1 molal solution. For RbCl (Figure 7b), the simulations and experiments exhibit the same initial drop, but afterward they both decrease more slowly up to 4 molal. The initial drop in the activity coefficients for both salts is a direct result of the leading m_s_^1/2^ dependence dictated by the Debye–Hückel limiting law. This drop is more significant for the simulations as a result of the dielectric constant for TIP4*P*/2005 water (ε = 59.1) being lower than that for real water (ε = 78.3^56^), resulting in a greater value of *Ã* as dictated by eq 11 for TIP4*P*/2005 water. More interestingly, the simulation results for both NaBr and RbCl are shifted downward from the experiment by 0.1 to 0.2 at higher concentrations. The deeper minimum in the activity coefficient could be remedied by either using a water model that exhibits the correct dielectric constant for water or by using scaled-ion charges to mimic dielectric screening effects as has been proposed by a number of investigators.^26,54,57,58^ The fits of the modDH model at ambient pressure (eq 18) to the osmotic pressures determined using the optimized interactions above are reported in Table 2. Fits for simulations using the unmodified Lennard-Jones interactions (χ = 0) are reported in the Supporting Information (Table S3).

## Conclusions

Here, we utilized osmotic force balance simulations for measuring the osmotic pressures of ions in solution to examine the osmotic pressures of a wide range of alkali halide salts up to concentrations of ∼4 M. The utility of the osmotic force balance technique is that the osmotic pressure over a wide range of concentrations can be evaluated from a single simulation. Moreover, the application of an external potential applied to the ions to perform these simulations does not require specialized coding of the available molecular simulation packages. The cation and anion Lennard-Jones cross-interaction parameters were optimized to reproduce the experimental osmotic pressures of all of the simulated salts. We were able to fit the concentration profiles obtained from the osmotic force balance simulations utilizing an assumed form for the free energies of ions in solution, the modified Debye–Hückel equation. This theory quantitively described the salt concentration profiles from low to high concentrations, in addition to providing an analytical expression for the salt osmotic pressures. We also provided a framework for mapping osmotic force balance simulation results to measurements of the activity of water that is typically determined experimentally at ambient pressures, thereby facilitating an improved comparison between simulations and experiment.

Accurate force field parameters are essential for meaningful simulation studies of molecular association in solution. Force fields, however, are frequently only parametrized to reproduce solute properties at infinite dilution. The framework laid out here is not limited to monovalent electrolyte solutions but can be applied to a broader range of systems. For example, it has been noted that interactions for both monovalent^59,60^ and multivalent^25^ salts can have erroneous aggregation propensities, confounding predictions of charge-mediated properties in solution. Fitting ion potentials to properties such as the osmotic pressure could thereby potentially improve the description of salts at concentrations close to their solubility limits. Indeed, using an off the shelf force field for the fluoride ion here proved problematic, resulting from exaggerated solute aggregation. Following the work of Vega and co-workers,^26^ it would potentially be useful to also fit additional solution properties like the salt solution density, which could also necessitate adjustments of ion–water interactions as well. Beyond electrolyte solutions, the osmotic force balance technique could also be applied to systems like hydrophobes in water, where a knowledge of the osmotic pressure could provide insights into the thermodynamic driving forces for assembly.^61−63^

## Supporting Information Available

Details for the osmotic force balance simulations and isothermal–isobaric ensemble simulations; fits of the salt chemical potentials for the simulations using the unmodified (χ = 0) Lennard-Jones cross interactions to the modDH expressions at constant μ_w_ and *P*; fits of the salt solution densities at constant μ_w_ and *P* for the simulations using the unmodified (χ = 0) Lennard-Jones cross interactions to eq 22; simulation and fitted concentration profiles from the osmotic force balance simulations using the unmodified (χ = 0) and optimized Lennard-Jones cross interactions for all the simulated salts except NaBr and RbCl; osmotic pressures determined from the osmotic force balance simulations using the unmodified (χ = 0) and optimized Lennard-Jones cross interactions for all the simulated salts except NaBr and RbCl; and activity coefficients of the salts at atmospheric pressure using the optimized Lennard-Jones cross interactions for all the simulated salts except NaBr and RbCl (PDF)
