# Supplementary material for: Osmotic Force Balance Evaluation of Aqueous Electrolyte Osmotic Pressures and Chemical Potentials
Source: J Chem Theory Comput. 2023 Nov 18;19(23):8826–38. doi: 10.1021/acs.jctc.3c00982 (PMC10720338; doi:10.1021/acs.jctc.3c00982)
Supplement: Supplementary file 1 — ct3c00982_si_001.pdf [file ct3c00982_si_001.pdf]

Supporting Information for

Osmotic Force Balance Evaluation of Aqueous Electrolyte Osmotic Pressures and Chemical Potentials

Alireza Hosseni[1] and Henry S. Ashbaugh[1,*]

[1]Department of Chemical and Biomolecular Engineering, Tulane University, New Orleans, LA 70118

In this supplement we provide additional information regarding our simulations to determine the osmotic pressure of salts in solution. We detail the contents of this supplement below.

In Table S1 (page S3) we provide details of the osmotic force balance simulations (*e.g.*, numbers of waters, ions, and box dimensions) performed to evaluate the osmotic equations-of-state of the original ($\chi = 0$) and optimized ($\chi =$ optimal) cross interactions. The temperature was set to 25°C. These simulations were equilibrated for 25 ns, followed by production runs of 200 ns for evaluation of thermodynamic averages.

In Table S2 (page S4) we provide details of the isothermal-isobaric ensemble simulations of LiI, KCl, and CsBr at concentrations of 1, 2, and 3 molal using the optimized cross interactions. These temperature and pressure in these simulations were 25°C and 1 bar, respectively. These simulations were equilibrated for 25 ns, followed by production runs of 200 ns for evaluation of thermodynamic averages.

*corresponding author. email: hanka@tulane.edu

In Table S3 (page S5) we provide the fittings of the modDH expressions for the salt activity coefficients (eqs. (10) and (18)) to the simulations conducted with the original ($\chi = 0$) salt cross interactions.

In Table S4 (page S6) we provide fittings of eq. (22) to the simulation densities at constant μ_w and P conducted with the original ($\chi = 0$) salt cross interactions.

Figures S1 – S13 (pages S7 – S19) report the simulation concentration profiles along the z-axis of the 30 Å x 30 Å x 100 Å box for the salts LiCl, LiBr, LiI, NaCl, NaI, KCl, KBr, KI, RbBr, RbI, CsCl, CsBr, and CsI using the original ($\chi = 0$) and optimized ($\chi =$ optimal) cross interactions.

Figures S14 – S26 (pages S20 – S32) report the osmotic pressures determined using the the original ($\chi = 0$) and optimized ($\chi =$ optimal) cross interactions for the salts LiCl, LiBr, LiI, NaCl, NaI, KCl, KBr, KI, RbBr, RbI, CsCl, CsBr, and CsI.

Figures S27 – S39 (pages S33 – S45) report the salt activity coefficients described by eq. (18) for the salts LiCl, LiBr, LiI, NaCl, NaI, KCl, KBr, KI, RbBr, RbI, CsCl, CsBr, and CsI fitted to the simulations using the optimized ($\chi =$ optimal) cross interactions.

Table S1. Details for the osmotic force balance simulations. The box dimensions L_x, L_x, and L_x are reported in units of Å, while the external harmonic spring constant k has units of kJ/(mol Å^2).

salt	χ	L_x and L_y	L_z	N_w	N_s	k
LiCl	0	30	100	2928	60	0.025
	0.75	30	100	2892	60	0.025
LiBr	0	30	100	2896	60	0.025
	0.9	30	100	2848	60	0.025
LiI	0	30	100	2843	60	0.03
	1.06	30	100	2777	60	0.03
NaCl	0	30	100	2951	60	0.025
	-0.32	30	100	2978	60	0.025
NaBr	0	30	100	2922	60	0.025
	-0.41	30	100	2960	60	0.025
	0	30	50	1461	30	0.1
	0	28.2311	28.2311	730	15	0.313679
NaI	0	30	100	2871	60	0.03
	-0.55	30	100	2938	60	0.03
KCl	0	30	100	2912	60	0.0225
	0.25	30	100	2892	60	0.0225
KBr	0	30	100	2879	60	0.0225
	-0.3	30	100	2910	60	0.0225
KI	0	30	100	2829	60	0.0225
	0.2	30	100	2810	60	0.0225
RbCl	0	30	100	2897	60	0.0225
	0.5	30	100	2856	60	0.0225
	0	30	50	1449	30	0.09
	0	28.2311	28.2311	724	15	0.282311
RbBr	0	30	100	2865	60	0.025
	0.43	30	100	2827	60	0.025
RbI	0	30	100	2814	60	0.025
	0.35	30	100	2778	60	0.025
CsCl	0	30	100	2875	60	0.0225
	0.34	30	100	2844	60	0.0225
CsBr	0	30	100	2842	60	0.025
	0.22	30	100	2820	60	0.025
CsI	0	30	100	2861	40	0.02

Table S2. Details for the isothermal-isobaric ensemble salt solution simulations.

salt	m_s	N_s	N_w
LiI	1.0003	51	2830
	2.0020	95	2634
	3.0005	134	2479
KCl	1.0005	52	2885
	2.0001	101	2803
	3.0005	146	2701
CsBr	1.0003	51	2830
	2.0001	97	2692
	3.0022	139	2570

Table S3. Parameters of the modified Debye-Hückel expressions for the free energy of the simulated salts in aqueous solution. Table **a)** reports fits determined directly from the osmotic force balance simulations at constant μ_w. The units of A, B, α_1, and α_2 are $M^{-1/2}$, $M^{-1/2}$, M^{-1}, and M^{-2}, respectively. Table **b)** reports fits to at constant P to the osmotic pressures. The units of $\tilde{A}$, $\tilde{B}$, $\tilde{\alpha}_1$, and $\tilde{\alpha}_2$ are molal$^{-1/2}$, molal$^{-1/2}$, molal^{-1}, and molal^{-2}, respectively.

a) Coefficients for $\mu_s(C_s|\mu_w)$ along a line of constant μ_w (eq. (10) with A = 1.7964).

salt	LiCl	LiBr	LiI	NaCl	NaBr	NaI	KCl	KBr
B	0.88131	1.4505	1.3390	1.9247	1.6519	1.7051	1.9562	0.88105
α_1	0.30466	0.27772	0.34922	9.3393×10^{-2}	0.27736	0.38438	5.8777×10^{-2}	0.28597
α_2	-3.5799×10^{-3}	1.1463×10^{-2}	3.6955×10^{-2}	3.4572×10^{-2}	2.1738×10^{-2}	4.6526×10^{-2}	1.4035×10^{-2}	-1.0100×10^{-2}

salt	KI	RbCl	RbBr	RbI	CsCl	CsBr	CsI
B	1.2649	1.7744	1.2926	1.2819	1.0502	1.4433	1.5623
α_1	0.30604	3.4415×10^{-2}	0.19667	0.34264	0.15247	0.15406	0.10900
α_2	-7.4104×10^{-3}	2.8867×10^{-2}	4.3776×10^{-3}	-1.6398×10^{-2}	1.0312×10^{-2}	7.3202×10^{-3}	-1.8300×10^{-3}

b) Coefficients for $\mu_s(m_s|P)$ along a line of constant P (eq. (18) with $\tilde{A}$ = 1.7937).

salt	LiCl	LiBr	LiI	NaCl	NaBr	NaI	KCl	KBr
$\tilde{B}$	0.87999	1.4483	1.3370	1.9218	1.6494	1.7025	1.9532	0.8797
$\tilde{\alpha}_1$	0.27075	0.22946	0.29867	9.0418×10^{-2}	0.25377	0.35760	2.5807×10^{-2}	0.21906
$\tilde{\alpha}_2$	-1.2375×10^{-2}	-4.0158×10^{-3}	-1.1822×10^{-3}	1.9887×10^{-2}	2.1804×10^{-3}	3.3642×10^{-3}	3.9211E-03	-1.7598×10^{-2}

salt	KI	RbCl	RbBr	RbI	CsCl	CsBr	CsI
$\tilde{B}$	1.2630	1.7718	1.2906	1.2799	1.0486	1.4411	1.5599
$\tilde{\alpha}_1$	0.20562	8.9628×10^{-3}	0.13404	0.21510	0.10398	8.0856×10^{-2}	3.5879×10^{-2}
$\tilde{\alpha}_2$	-1.8029×10^{-2}	1.1767×10^{-2}	-8.3090×10^{-3}	-2.2000×10^{-2}	-3.4012×10^{-3}	-5.2121×10^{-3}	-5.4064×10^{-3}

Table S4. Fits of eq. (22) to the simulation densities of aqueous salt solutions as a function of the salt molality along lines of constant μ_w and P at 25°C. The units of ρ_{w0}, θ_1^j, $\theta_{3/2}^j$, and θ_2^j are g/cm^3, g/(cm^3 molal), g/(cm^3 molal$^{3/2}$), and g/(cm^3 molal^2), respectively.

salt	LiCl	LiBr	LiI	NaCl	NaBr	NaI	KCl	KBr
ρ_{w0}	0.99706	0.99701	0.99673	0.99654	0.99715	0.99687	0.99711	0.99667
θ_1^μ	2.3157×10^{-2}	5.7418×10^{-2}	8.4905×10^{-2}	5.2650×10^{-2}	8.4267×10^{-2}	0.10960	5.7045×10^{-2}	8.9337×10^{-2}
$\theta_{3/2}^\mu$	-1.2979×10^{-3}	-2.1552×10^{-3}	-2.4100×10^{-4}	-6.1221×10^{-3}	-3.3828×10^{-3}	1.3848×10^{-3}	-6.6306×10^{-3}	-5.2852×10^{-3}
θ_2^μ	-4.6240×10^{-4}	-9.6060×10^{-4}	-2.7447×10^{-3}	-3.3110×10^{-5}	-1.5492×10^{-3}	-4.0864×10^{-3}	-4.0730×10^{-4}	-1.8148×10^{-3}
θ_1^P	2.1552×10^{-2}	5.5763×10^{-2}	8.3062×10^{-2}	5.0615×10^{-2}	8.2394×10^{-2}	0.10748	5.5268×10^{-2}	8.7915×10^{-2}
$\theta_{3/2}^P$	-1.2884×10^{-3}	-2.1127×10^{-3}	2.0892×10^{-4}	-5.3907×10^{-3}	-2.9494×10^{-3}	2.3231×10^{-3}	-6.5127×10^{-3}	-5.6067×10^{-3}
θ_2^P	-6.8390×10^{-4}	-1.3197×10^{-3}	-3.4189×10^{-3}	-6.3600×10^{-4}	-2.1979×10^{-3}	-5.1450×10^{-3}	-6.0270×10^{-4}	-1.9384×10^{-3}

salt	KI	RbCl	RbBr	RbI	CsCl	CsBr	CsI
ρ_{w0}	0.99676	0.99696	0.99728	0.99706	0.99728	0.99713	0.99679
θ_1^μ	0.11655	0.09965	0.13076	0.16111	0.14053	0.17494	0.22188
$\theta_{3/2}^\mu$	-3.2002×10^{-3}	-7.7413×10^{-3}	-5.4861×10^{-3}	-7.7788×10^{-3}	-8.9216×10^{-3}	-1.1128×10^{-2}	-6.8022×10^{-3}
θ_2^μ	-3.7961×10^{-3}	-1.3885×10^{-3}	-3.3988×10^{-3}	-4.5330×10^{-3}	-2.6780×10^{-3}	-3.7089×10^{-3}	-5.4566×10^{-3}
θ_1^P	0.11517	9.7580×10^{-2}	0.12924	0.16015	0.13876	0.17335	0.22035
$\theta_{3/2}^P$	-3.7333×10^{-3}	-7.0885×10^{-3}	-5.7092×10^{-3}	-8.9654×10^{-3}	-8.6804×10^{-3}	-1.1322×10^{-2}	-7.1660×10^{-3}
θ_2^P	-3.9021×10^{-3}	-1.8757×10^{-3}	-3.6286×10^{-3}	-4.4668×10^{-3}	-3.0533×10^{-3}	-3.9563×10^{-3}	-5.6528×10^{-3}

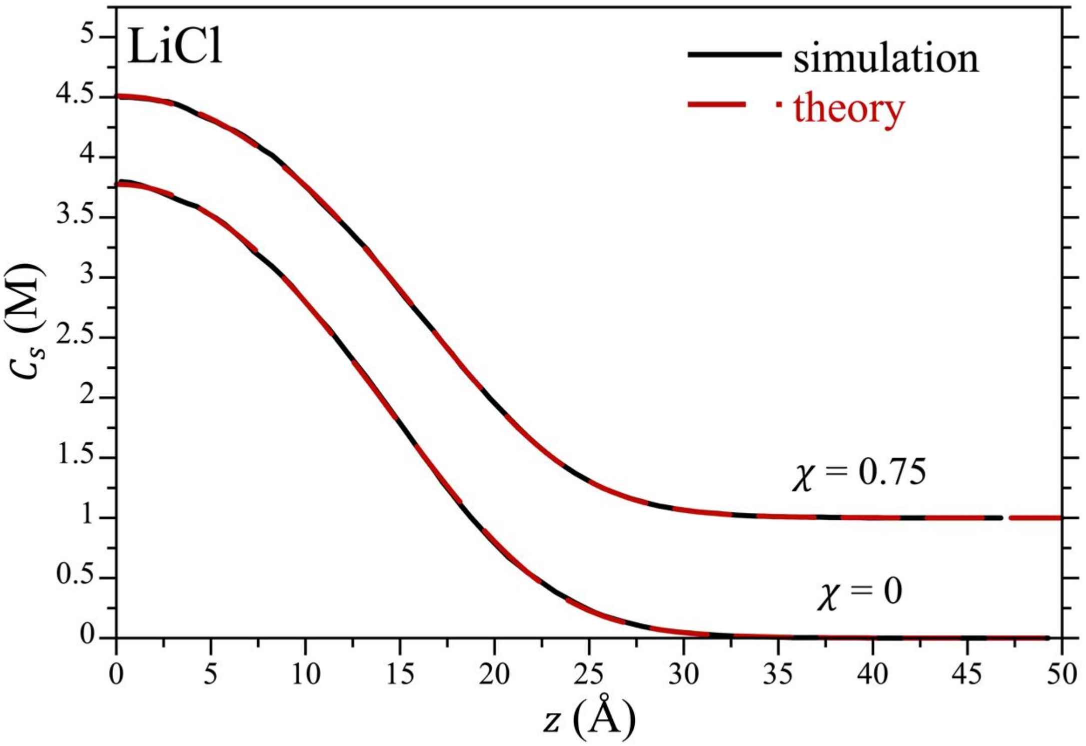

Figure S1. Comparison of the simulation concentration profiles as a function of position of LiCl against the fits of the modDH theoretical expression (eq. (10)). Results are reported for the unmodified ($\chi = 0$) and optimized ($\chi = 0.75$) potentials. The optimized potential results are shifted up by 1 M for clarity. The concentration profiles have been averaged about the line of symmetry (z.= 0) to improve statistical accuracy. The figure symbols are defined in the legend.

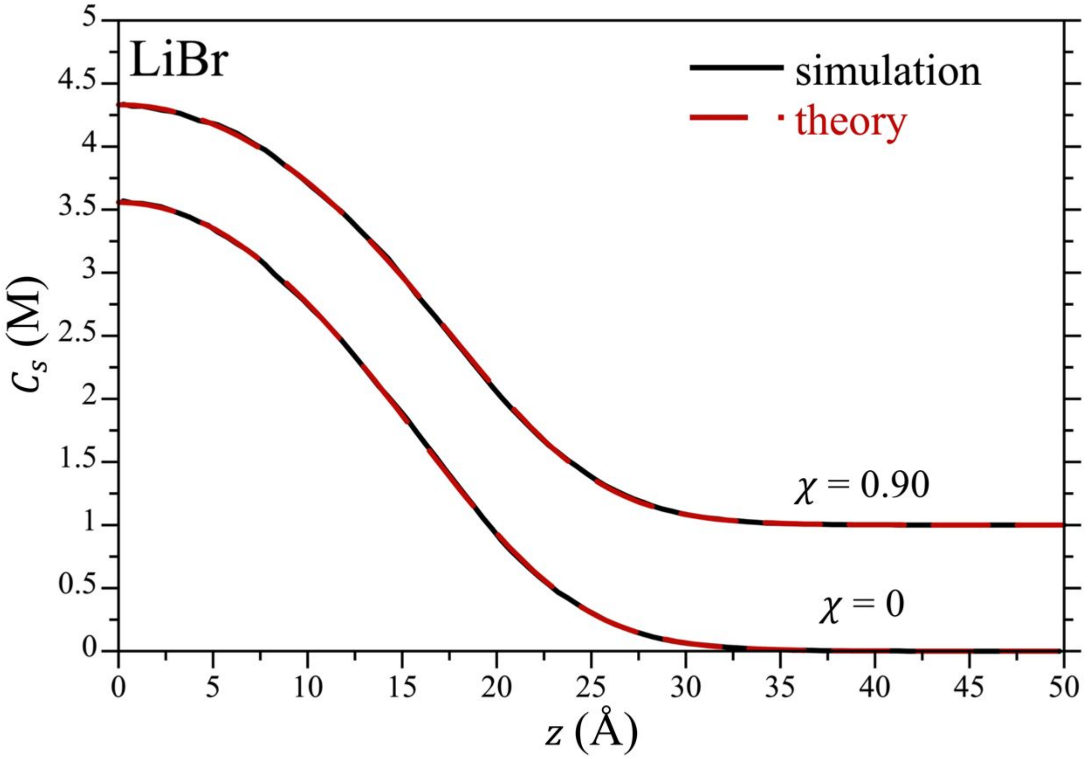

Figure S2. Comparison of the simulation concentration profiles as a function of position of LiBr against the fits of the modDH theoretical expression (eq. (10)). Results are reported for the unmodified ($\chi = 0$) and optimized ($\chi = 0.90$) potentials. The optimized potential results are shifted up by 1 M for clarity. The concentration profiles have been averaged about the line of symmetry (z.= 0) to improve statistical accuracy. The figure symbols are defined in the legend.

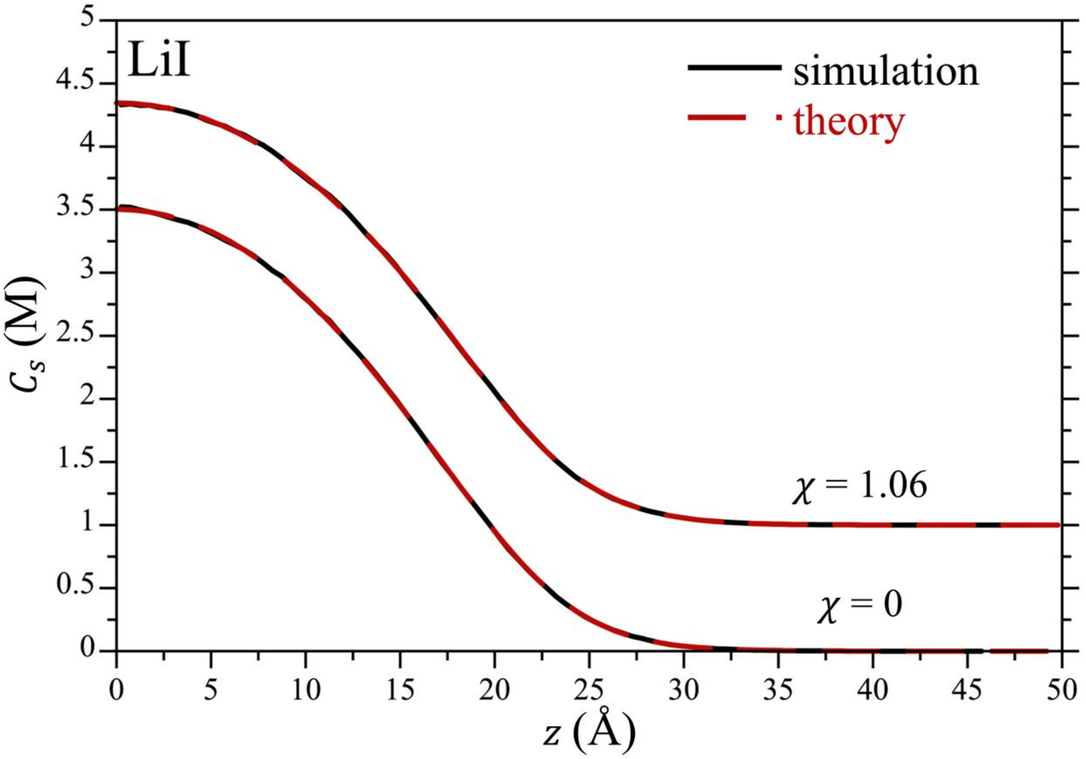

Figure S3. Comparison of the simulation concentration profiles as a function of position of LiI against the fits of the modDH theoretical expression (eq. (10)). Results are reported for the unmodified ($\chi = 0$) and optimized ($\chi = 1.06$) potentials. The optimized potential results are shifted up by 1 M for clarity. The concentration profiles have been averaged about the line of symmetry (z.= 0) to improve statistical accuracy. The figure symbols are defined in the legend.

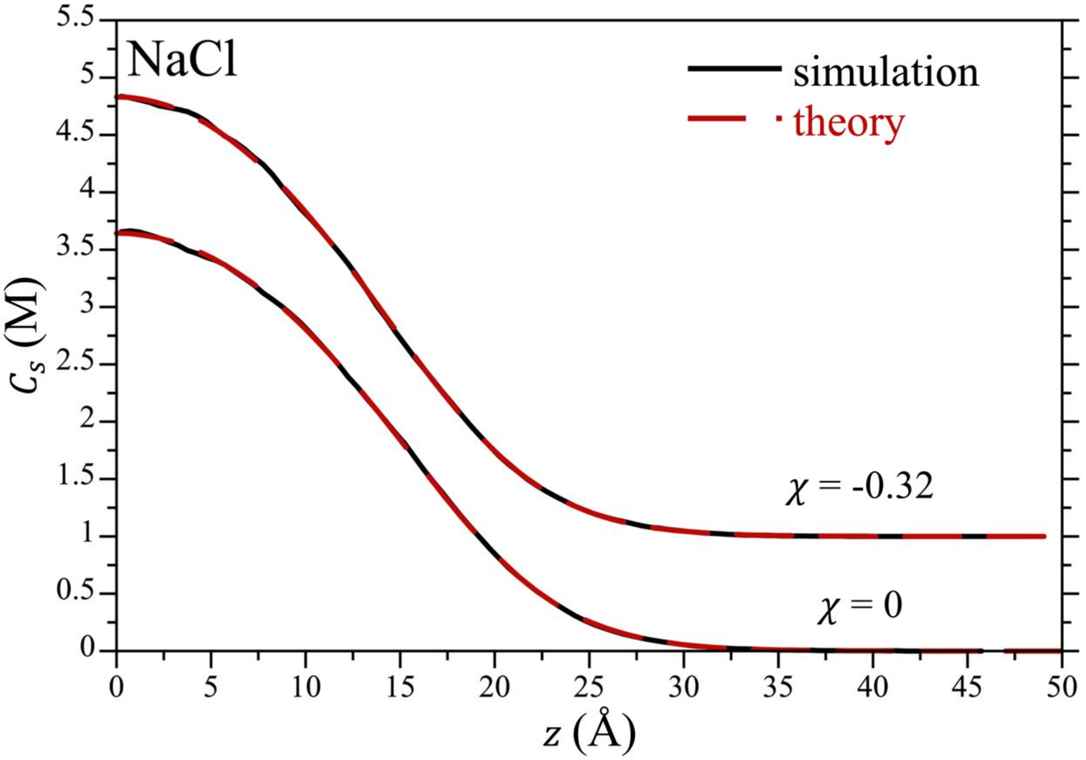

Figure S4. Comparison of the simulation concentration profiles as a function of position of NaCl against the fits of the modDH theoretical expression (eq. (10)). Results are reported for the unmodified ($\chi =$ 0) and optimized ($\chi =$ -0.32) potentials. The optimized potential results are shifted up by 1 M for clarity. The concentration profiles have been averaged about the line of symmetry (z.= 0) to improve statistical accuracy. The figure symbols are defined in the legend.

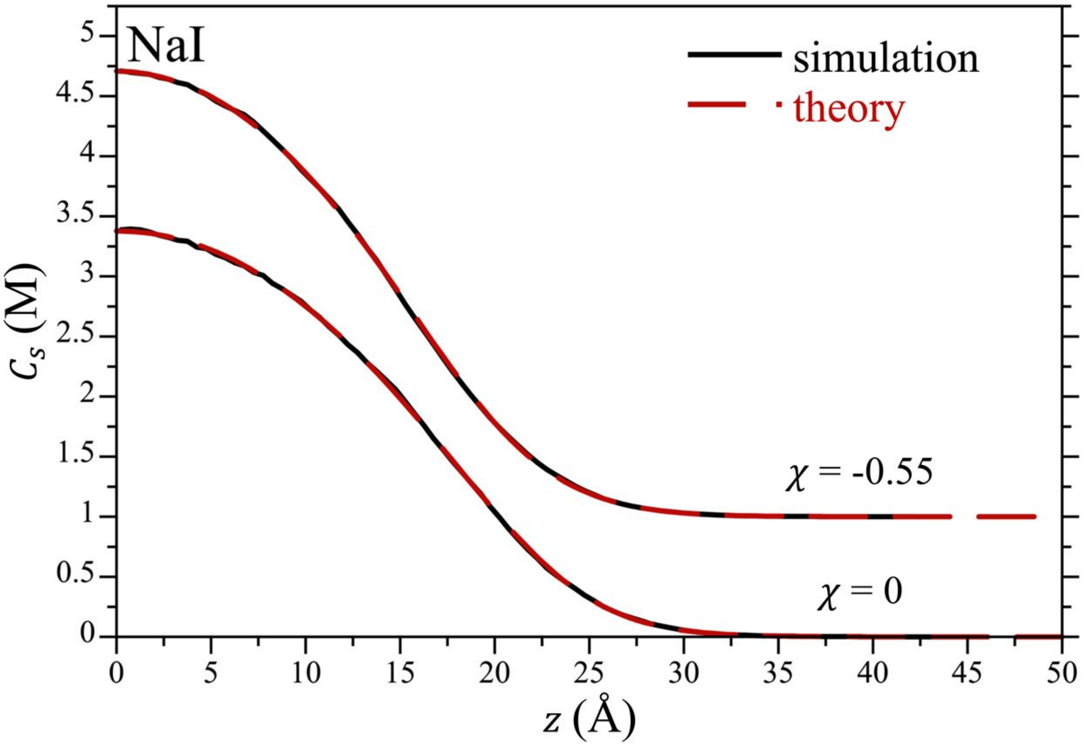

Figure S5. Comparison of the simulation concentration profiles as a function of position of NaI against the fits of the modDH theoretical expression (eq. (10)). Results are reported for the unmodified ($\chi = 0$) and optimized ($\chi = -0.55$) potentials. The optimized potential results are shifted up by 1 M for clarity. The concentration profiles have been averaged about the line of symmetry (z.= 0) to improve statistical accuracy. The figure symbols are defined in the legend.

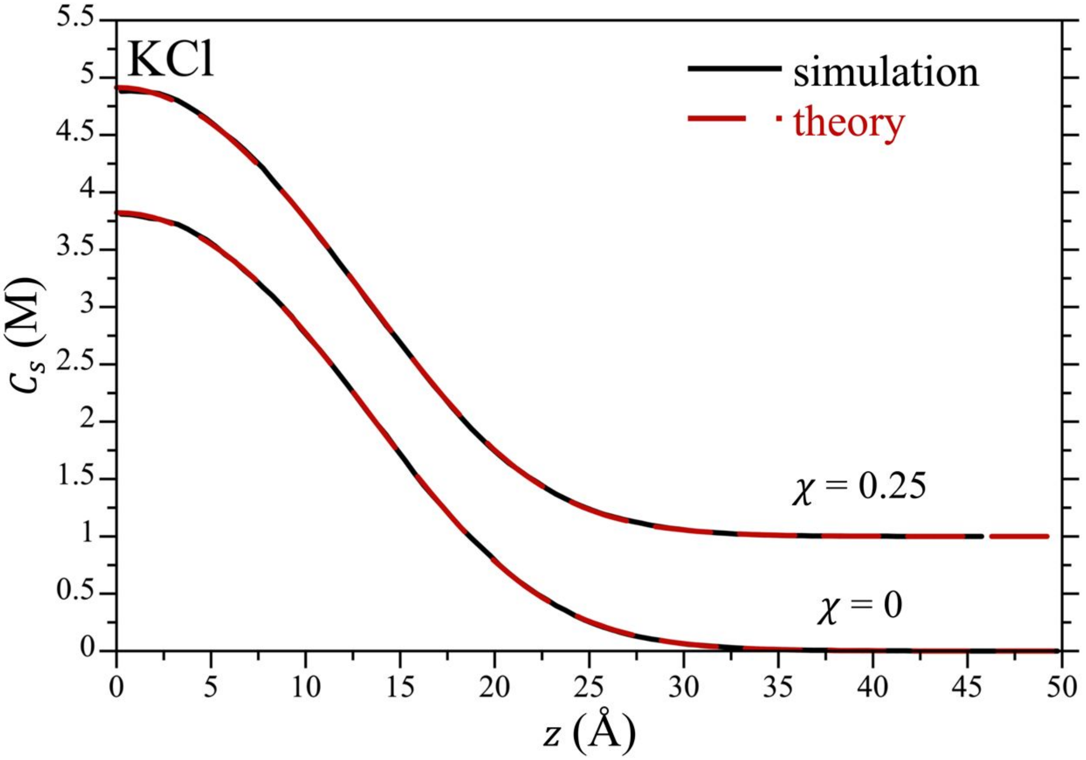

Figure S6. Comparison of the simulation concentration profiles as a function of position of KCl against the fits of the modDH theoretical expression (eq. (10)). Results are reported for the unmodified ($\chi = 0$) and optimized ($\chi = 0.25$) potentials. The optimized potential results are shifted up by 1 M for clarity. The concentration profiles have been averaged about the line of symmetry (z.= 0) to improve statistical accuracy. The figure symbols are defined in the legend.

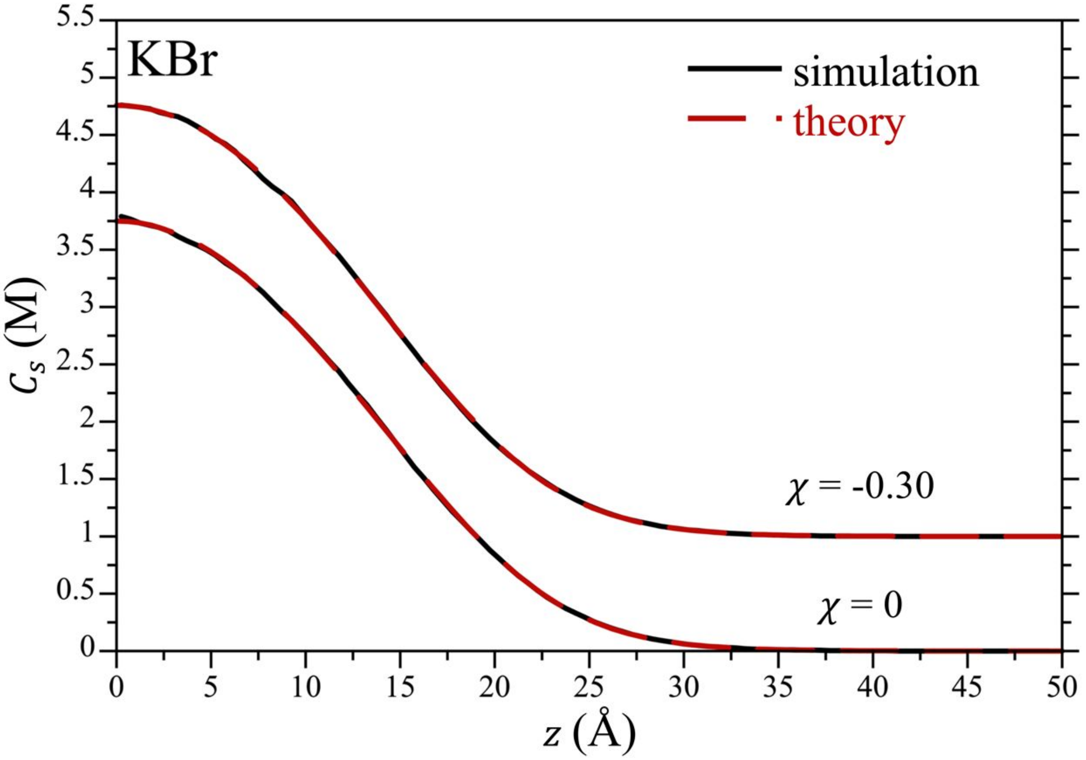

Figure S7. Comparison of the simulation concentration profiles as a function of position of KBr against the fits of the modDH theoretical expression (eq. (10)). Results are reported for the unmodified ($\chi = 0$) and optimized ($\chi = -0.30$) potentials. The optimized potential results are shifted up by 1 M for clarity. The concentration profiles have been averaged about the line of symmetry (z.= 0) to improve statistical accuracy. The figure symbols are defined in the legend.

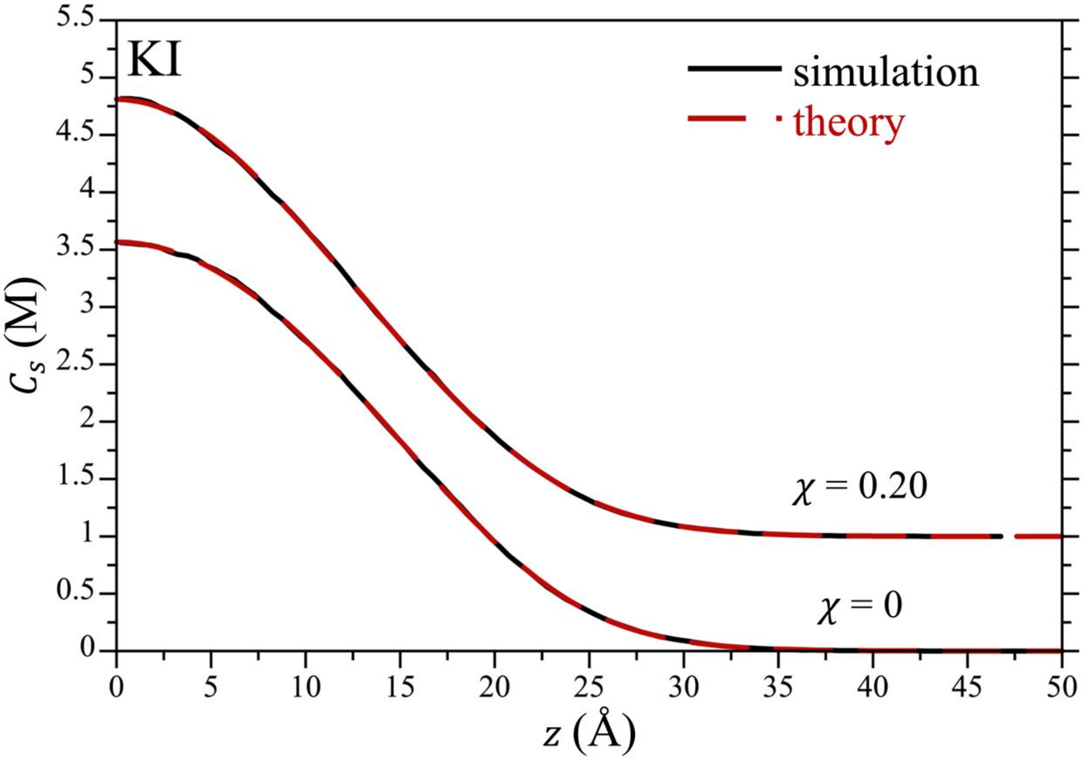

Figure S8. Comparison of the simulation concentration profiles as a function of position of KI against the fits of the modDH theoretical expression (eq. (10)). Results are reported for the unmodified ($\chi =\ 0$) and optimized ($\chi =\ 0.20$) potentials. The optimized potential results are shifted up by 1 M for clarity. The concentration profiles have been averaged about the line of symmetry (z.= 0) to improve statistical accuracy. The figure symbols are defined in the legend.

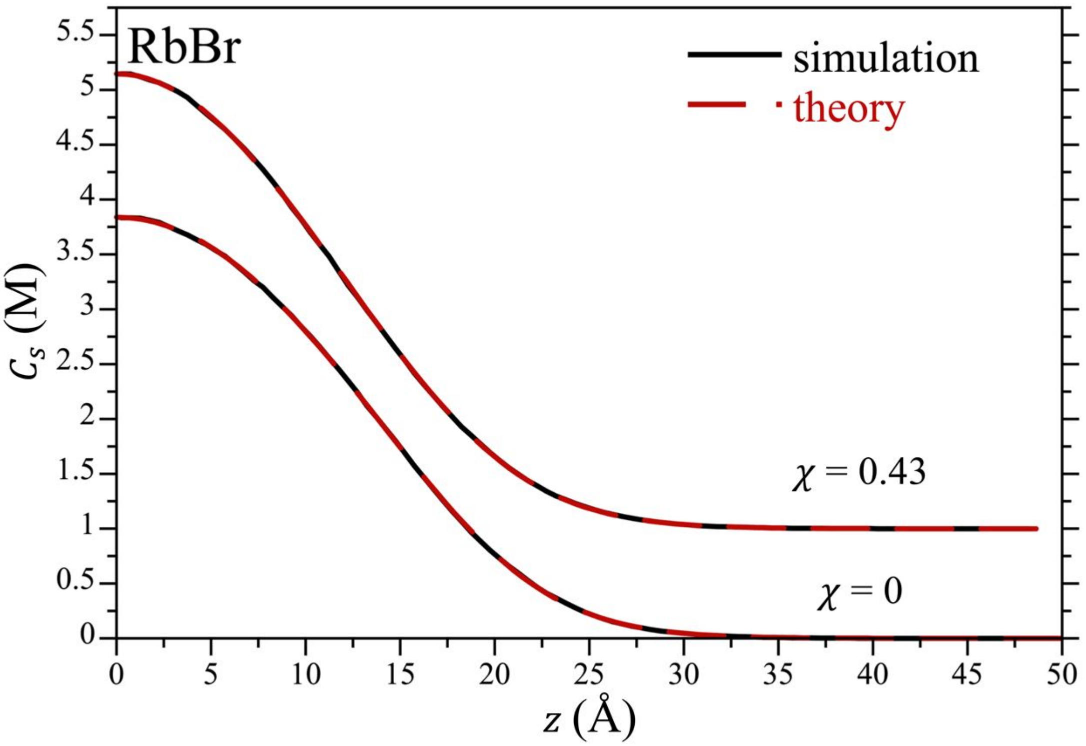

Figure S9. Comparison of the simulation concentration profiles as a function of position of RbBr against the fits of the modDH theoretical expression (eq. (10)). Results are reported for the unmodified ($\chi = 0$) and optimized ($\chi = 0.43$) potentials. The optimized potential results are shifted up by 1 M for clarity. The concentration profiles have been averaged about the line of symmetry (z.= 0) to improve statistical accuracy. The figure symbols are defined in the legend.

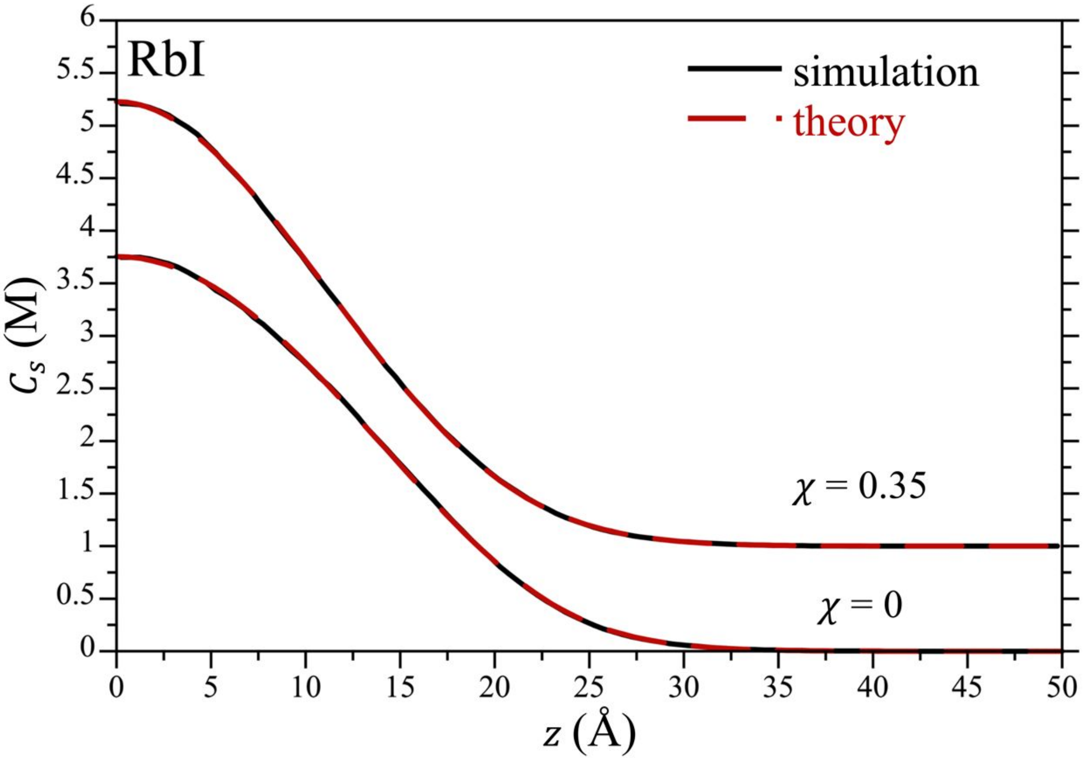

Figure S10. Comparison of the simulation concentration profiles as a function of position of RbI against the fits of the modDH theoretical expression (eq. (10)). Results are reported for the unmodified ($\chi = 0$) and optimized ($\chi = 0.35$) potentials. The optimized potential results are shifted up by 1 M for clarity. The concentration profiles have been averaged about the line of symmetry (z.= 0) to improve statistical accuracy. The figure symbols are defined in the legend.

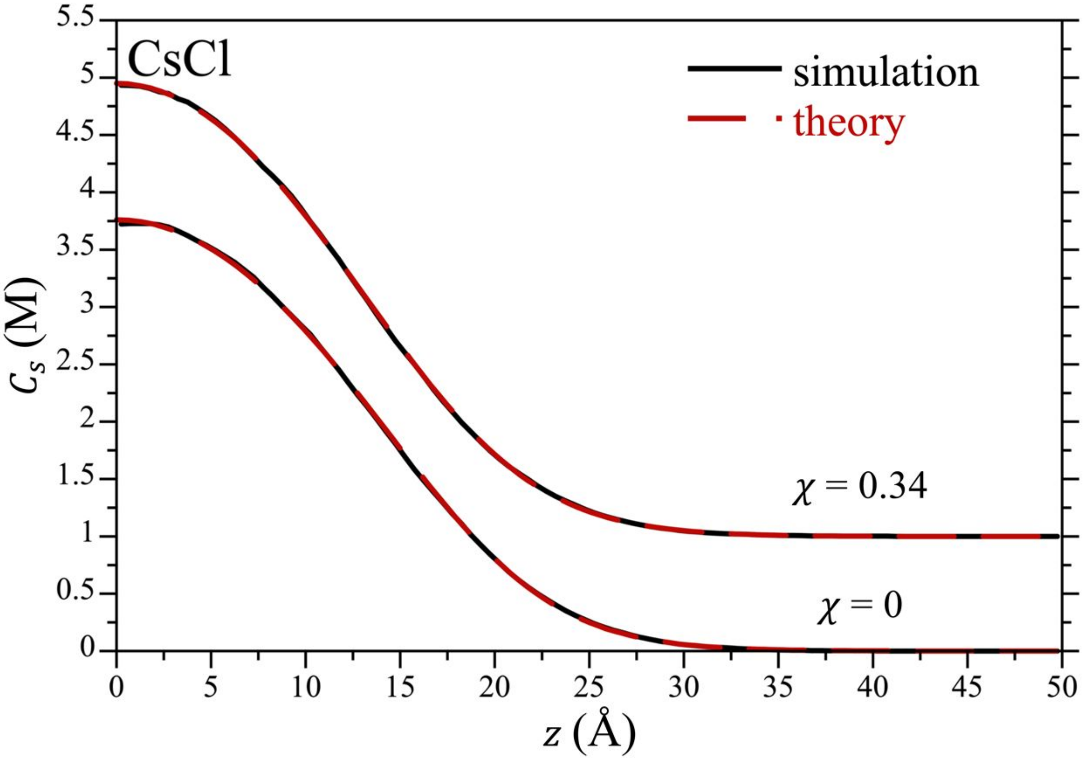

Figure S11. Comparison of the simulation concentration profiles as a function of position of CsCl against the fits of the modDH theoretical expression (eq. (10)). Results are reported for the unmodified ($\chi = 0$) and optimized ($\chi = 0.34$) potentials. The optimized potential results are shifted up by 1 M for clarity. The concentration profiles have been averaged about the line of symmetry (z.= 0) to improve statistical accuracy. The figure symbols are defined in the legend.

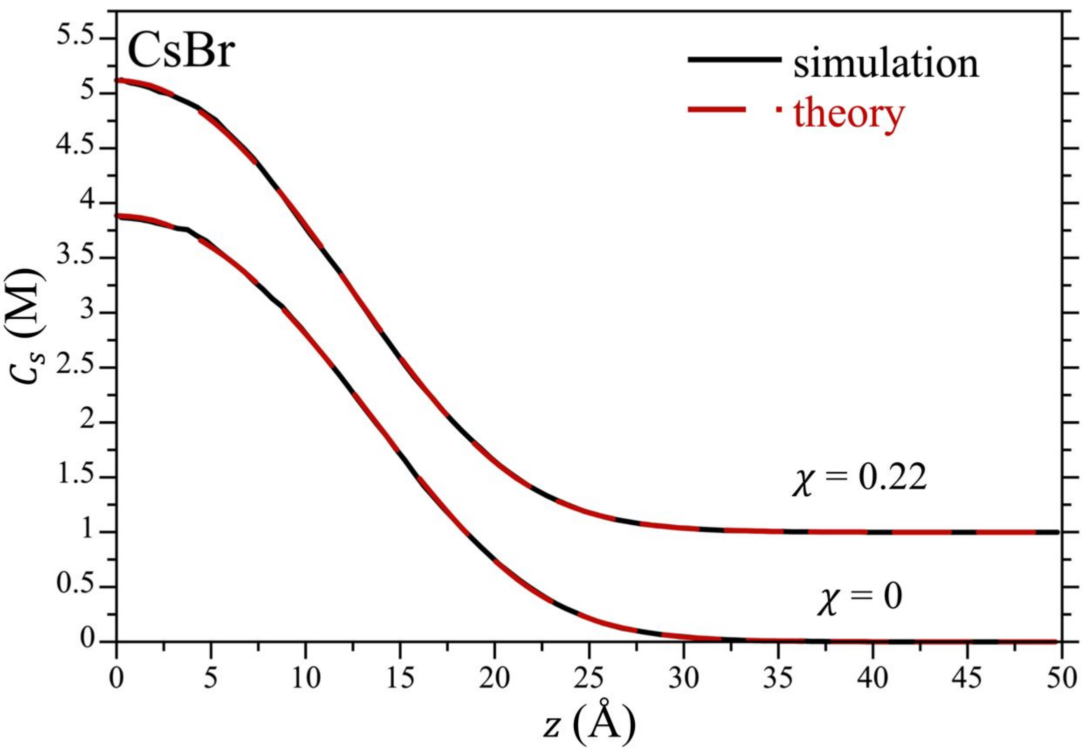

Figure S12. Comparison of the simulation concentration profiles as a function of position of CsBr against the fits of the modDH theoretical expression (eq. (10)). Results are reported for the unmodified ($\chi = 0$) and optimized ($\chi = 0.22$) potentials. The optimized potential results are shifted up by 1 M for clarity. The concentration profiles have been averaged about the line of symmetry (z.= 0) to improve statistical accuracy. The figure symbols are defined in the legend.

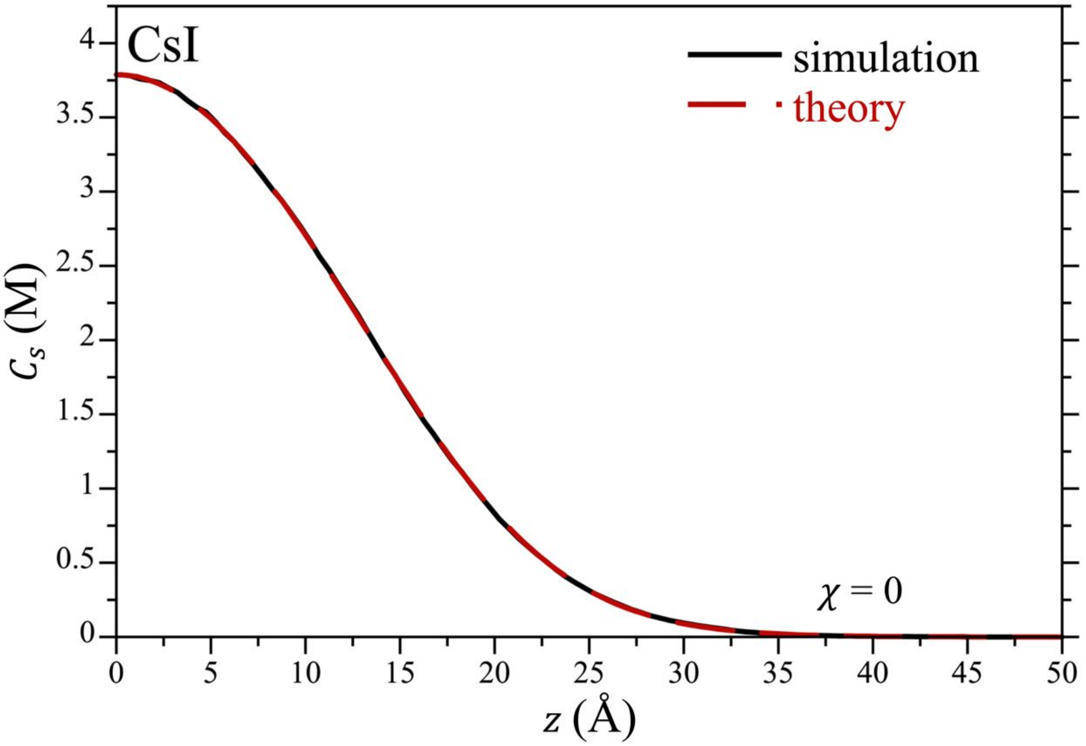

Figure S13. Comparison of the simulation concentration profiles as a function of position of CsI against the fits of the modDH theoretical expression (eq. (10)). Results are reported for the unmodified/optimized ($\chi =\ 0$) potential. The concentration profiles have been averaged about the line of symmetry (z.= 0) to improve statistical accuracy. The figure symbols are defined in the legend.

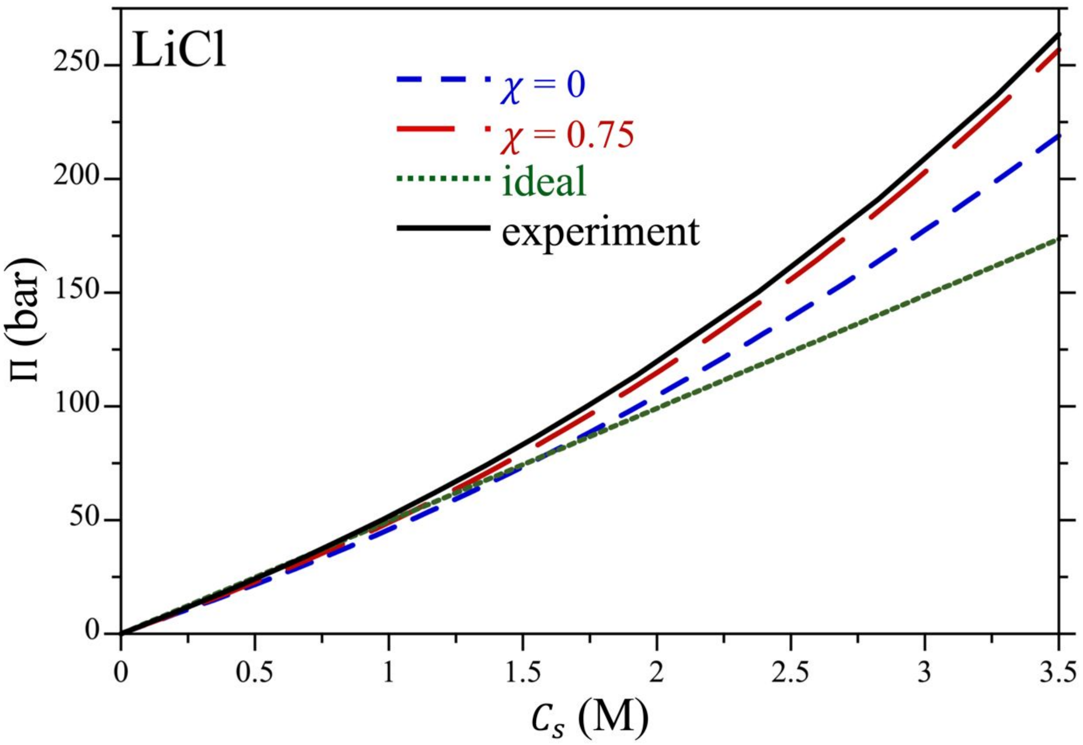

Figure S14. Osmotic pressure of LiCl in water as a function of the salt concentration. Results obtained from simulation using original ($\chi = 0$) and optimized ($\chi = 0.75$) potentials are compared against experiment and the ideal gas law. The experimental results are taken from Hammer and Wu.[1] The figure symbols are defined in the legend.

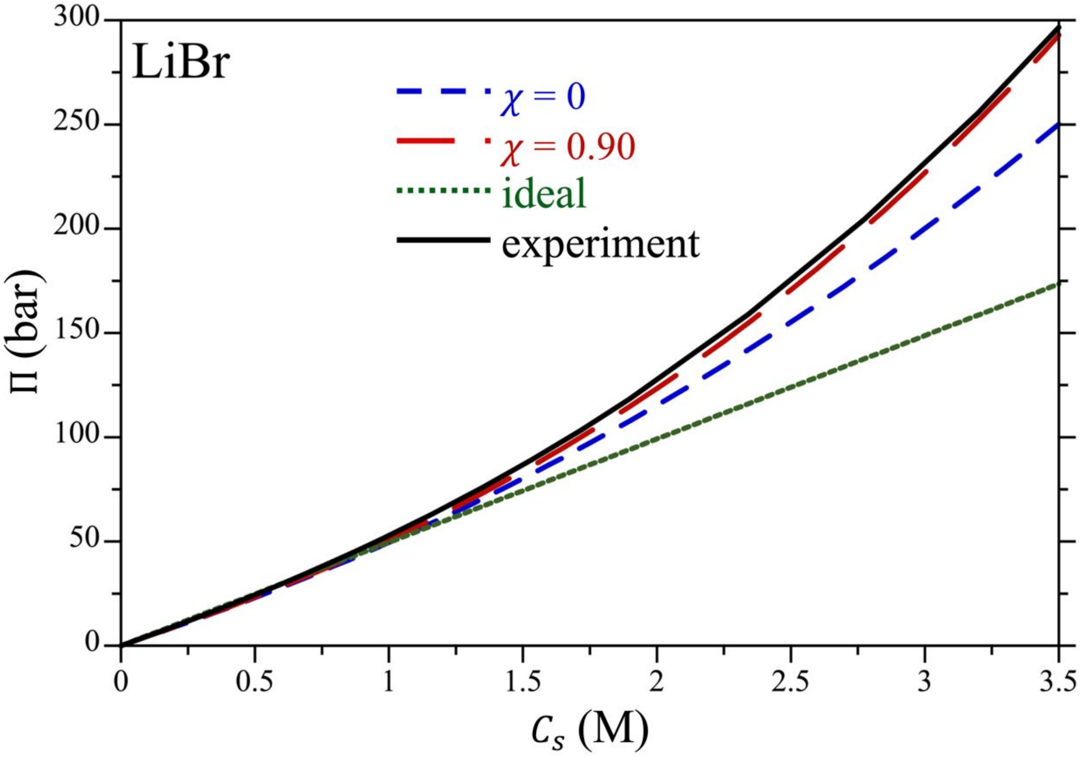

Figure S15. Osmotic pressure of LiBr in water as a function of the salt concentration. Results obtained from simulation using original ($\chi = 0$) and optimized ($\chi = 0.90$) potentials are compared against experiment and the ideal gas law. The experimental results are taken from Hammer and Wu.[1] The figure symbols are defined in the legend.

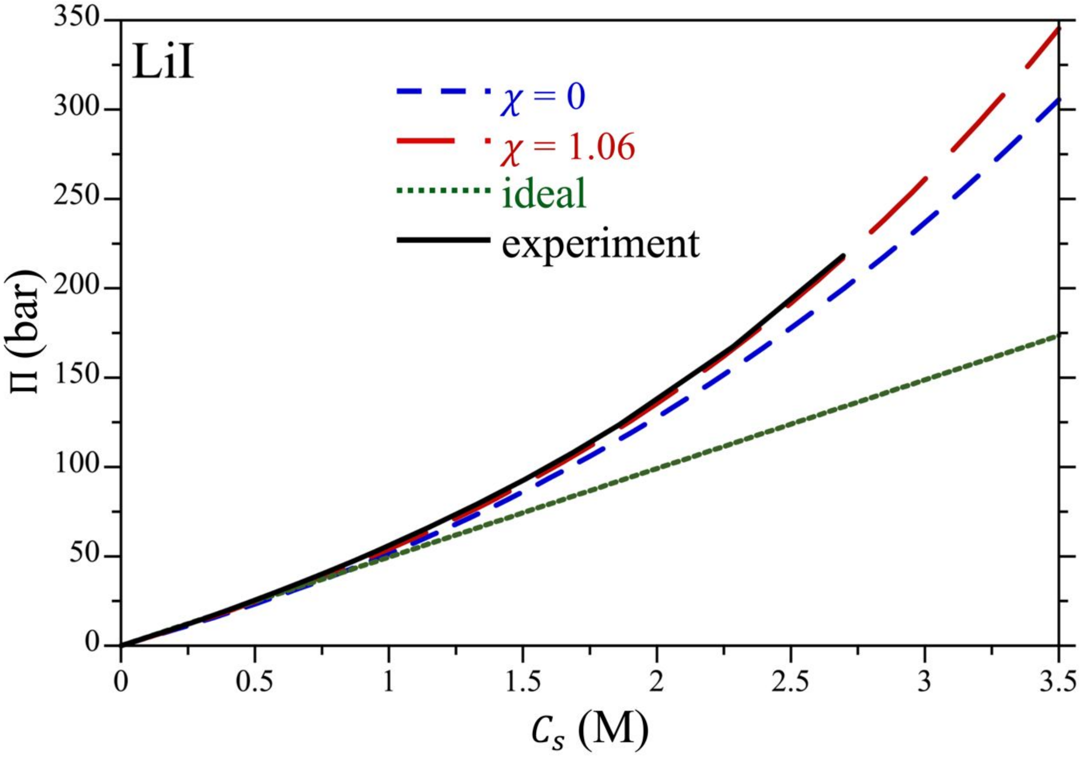

Figure S16. Osmotic pressure of LiI in water as a function of the salt concentration. Results obtained from simulation using original ($\chi = 0$) and optimized ($\chi = 1.06$) potentials are compared against experiment and the ideal gas law. The experimental results are taken from Hammer and Wu.[1] The figure symbols are defined in the legend.

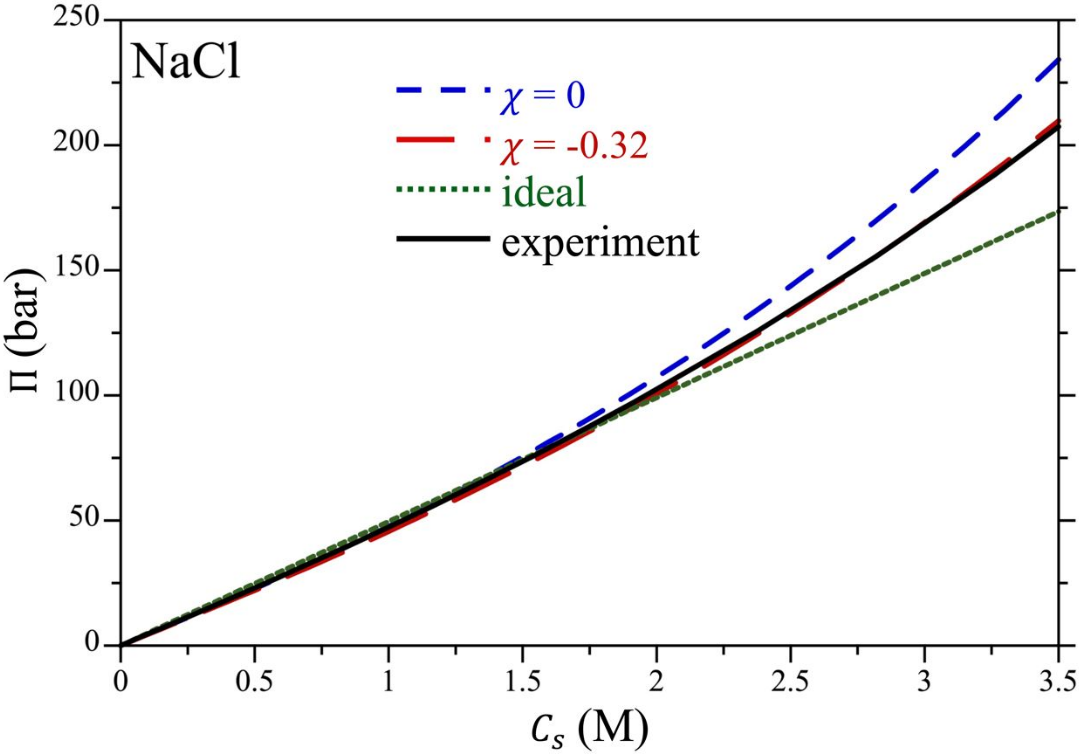

Figure S17. Osmotic pressure of NaCl in water as a function of the salt concentration. Results obtained from simulation using original ($\chi =$ 0) and optimized ($\chi =$ -0.32) potentials are compared against experiment and the ideal gas law. The experimental results are taken from Hammer and Wu.[1] The figure symbols are defined in the legend.

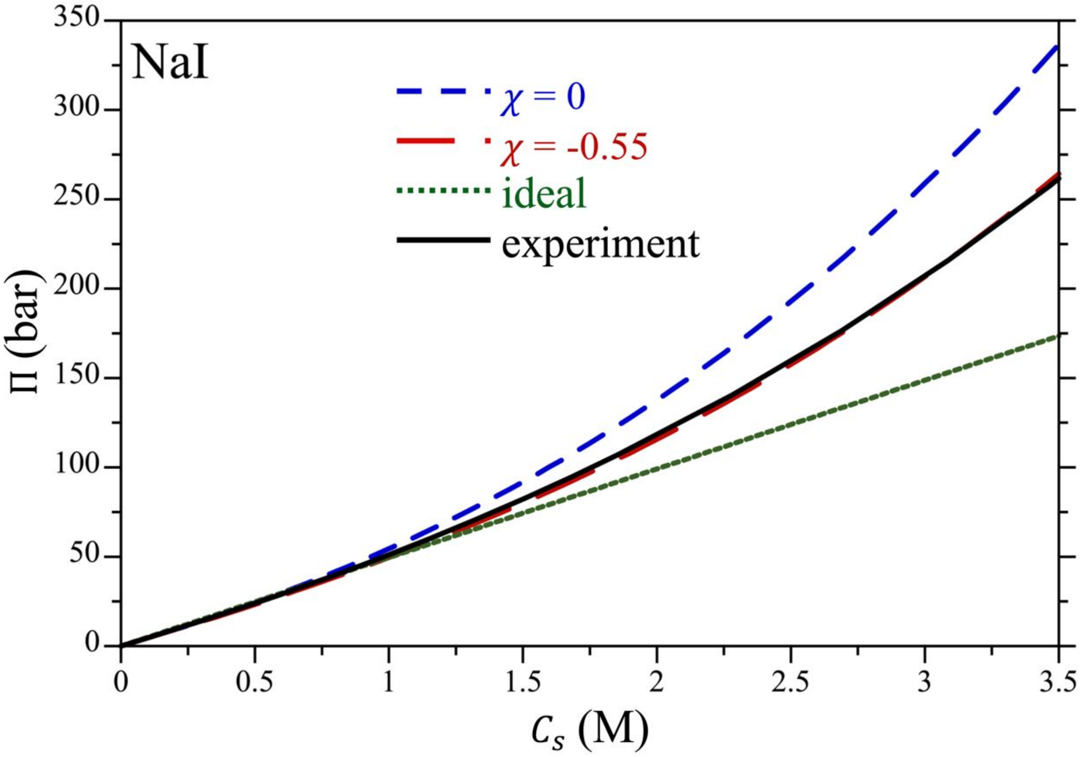

Figure S18. Osmotic pressure of NaI in water as a function of the salt concentration. Results obtained from simulation using original ($\chi =$ 0) and optimized ($\chi =$ -0.55) potentials are compared against experiment and the ideal gas law. The experimental results are taken from Hammer and Wu.[1] The figure symbols are defined in the legend.

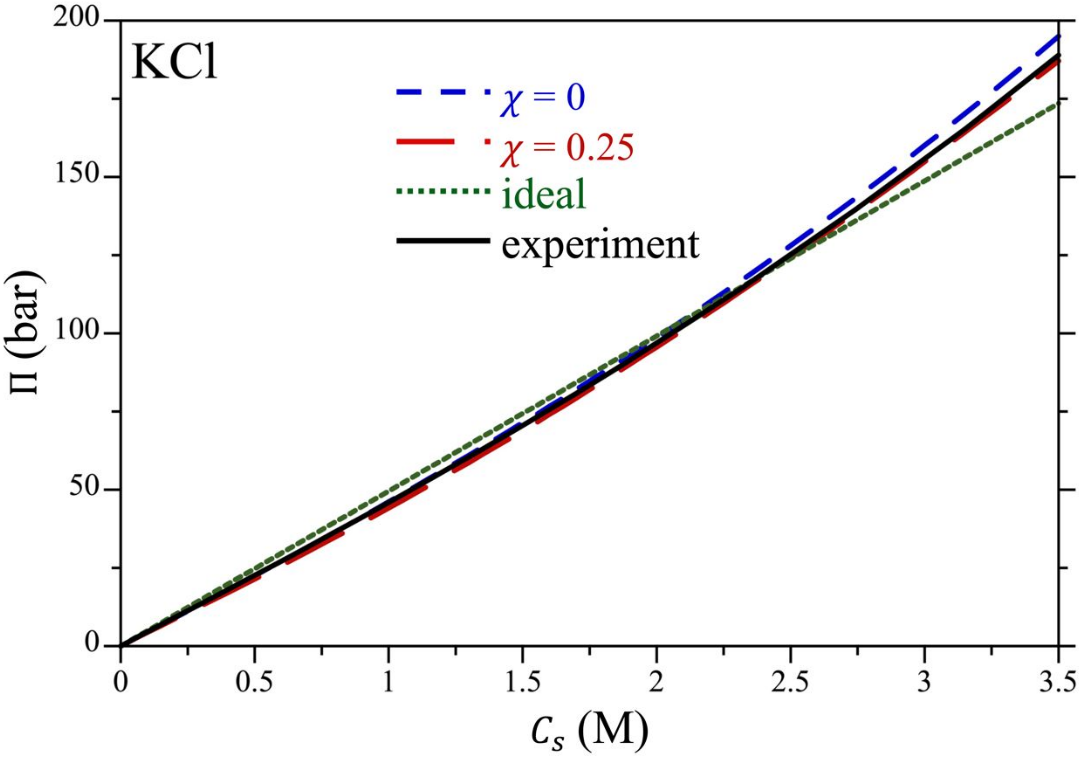

Figure S19. Osmotic pressure of KCl in water as a function of the salt concentration. Results obtained from simulation using original ($\chi = 0$) and optimized ($\chi = 0.25$) potentials are compared against experiment and the ideal gas law. The experimental results are taken from Hammer and Wu.[1] The figure symbols are defined in the legend.

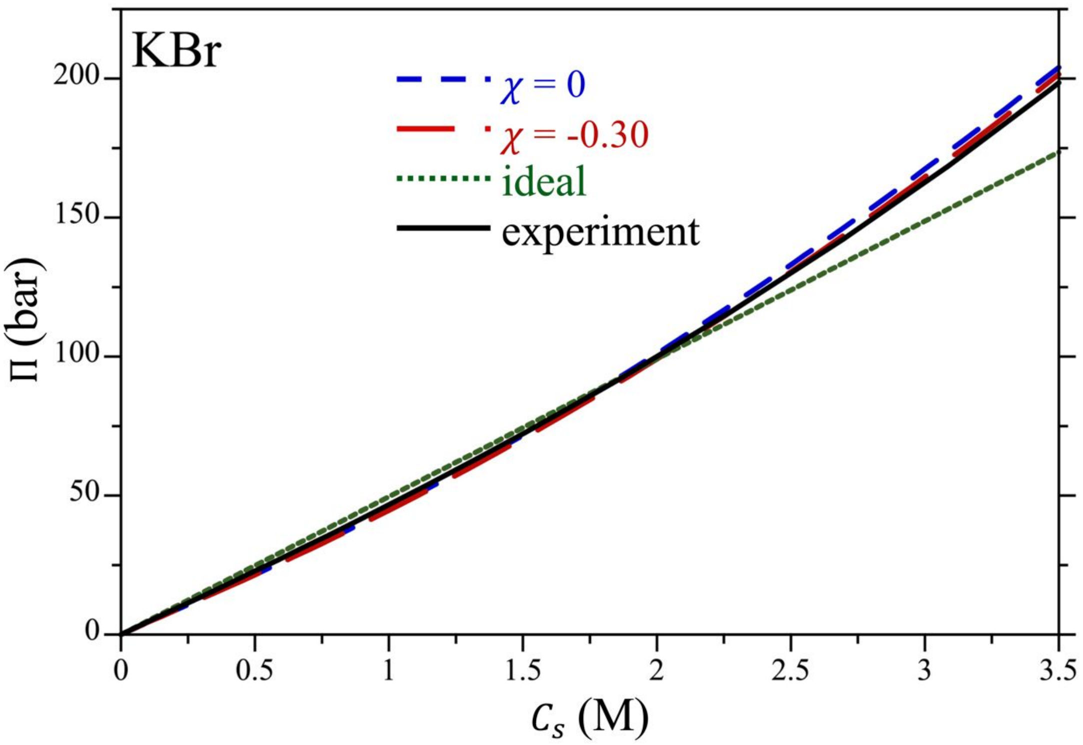

Figure S20. Osmotic pressure of KBr in water as a function of the salt concentration. Results obtained from simulation using original ($\chi =$ 0) and optimized ($\chi =$ -0.30) potentials are compared against experiment and the ideal gas law. The experimental results are taken from Hammer and Wu.[1] The figure symbols are defined in the legend.

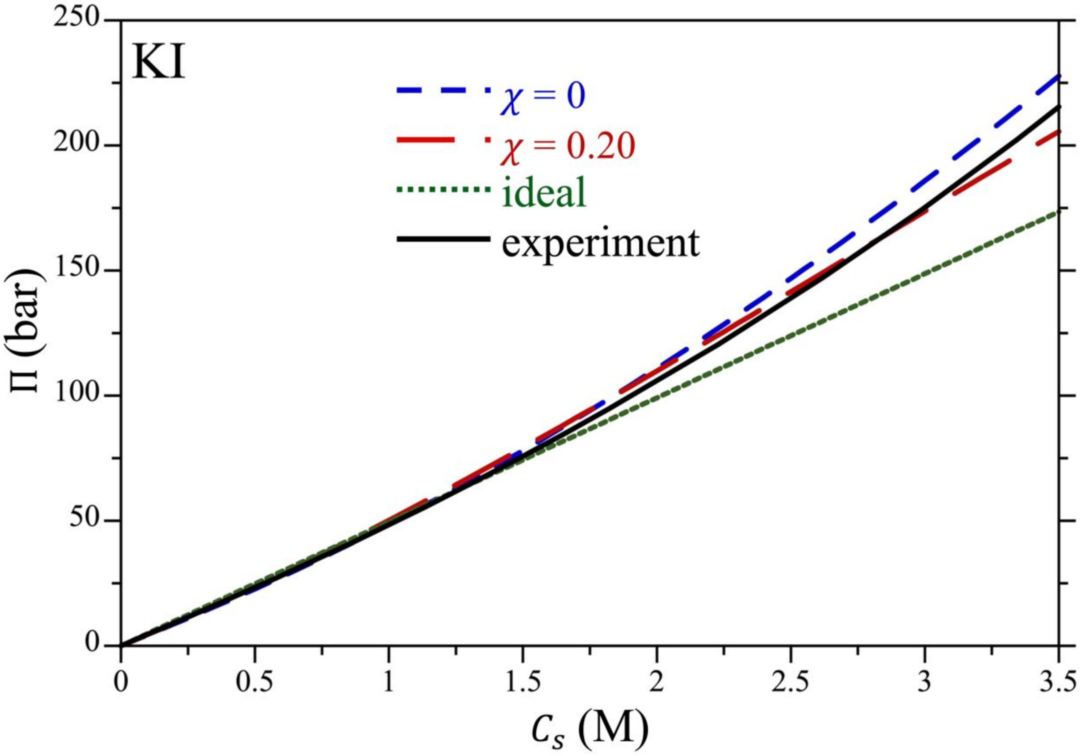

Figure S21. Osmotic pressure of KI in water as a function of the salt concentration. Results obtained from simulation using original ($\chi = 0$) and optimized ($\chi = 0.20$) potentials are compared against experiment and the ideal gas law. The experimental results are taken from Hammer and Wu.[1] The figure symbols are defined in the legend.

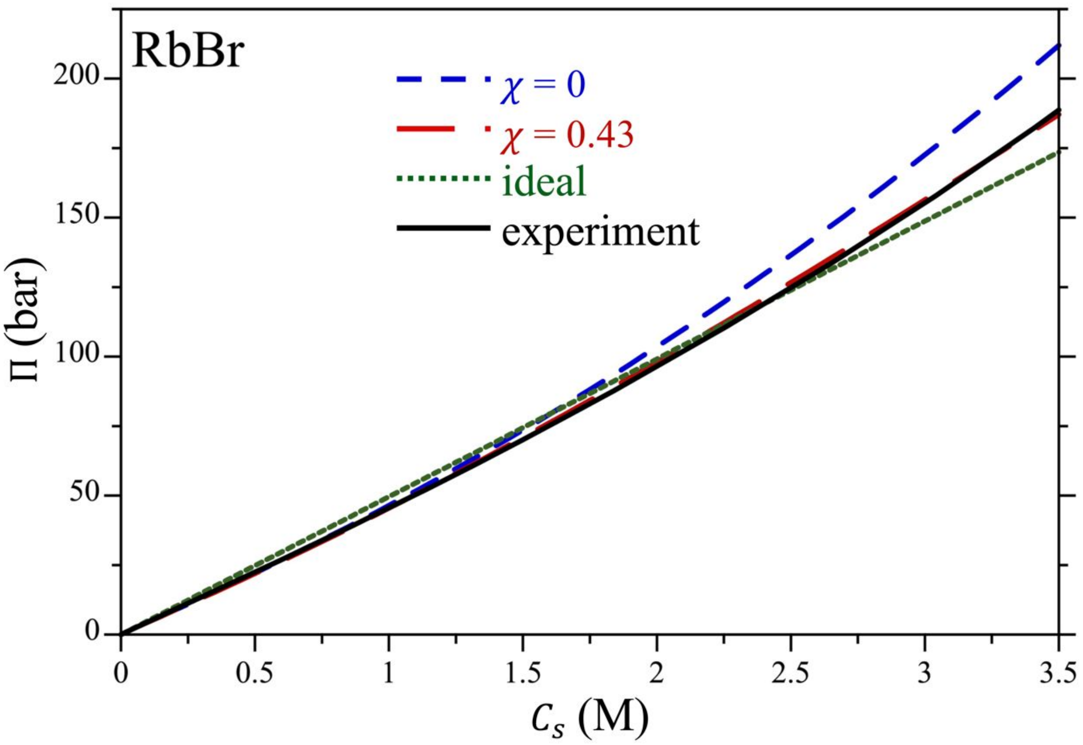

Figure S22. Osmotic pressure of RbBr in water as a function of the salt concentration. Results obtained from simulation using original ($\chi = 0$) and optimized ($\chi = 043$) potentials are compared against experiment and the ideal gas law. The experimental results are taken from Hammer and Wu.[1] The figure symbols are defined in the legend.

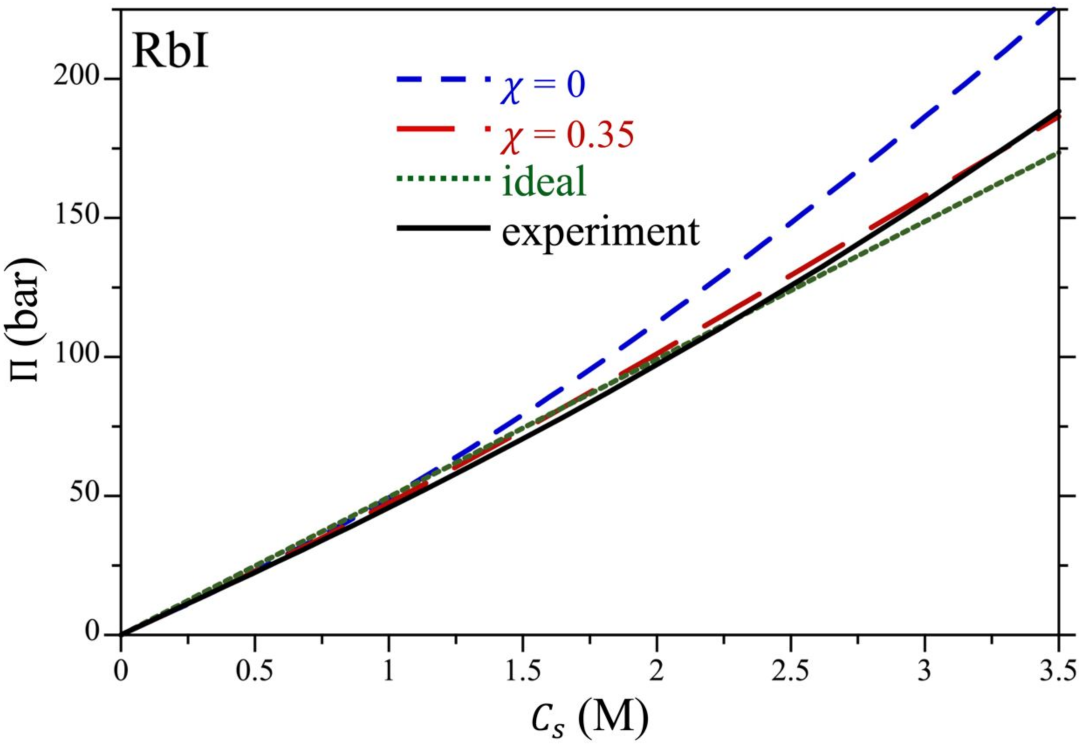

Figure S23. Osmotic pressure of RbI in water as a function of the salt concentration. Results obtained from simulation using original ($\chi = 0$) and optimized ($\chi = 0.35$) potentials are compared against experiment and the ideal gas law. The experimental results are taken from Hammer and Wu.[1] The figure symbols are defined in the legend.

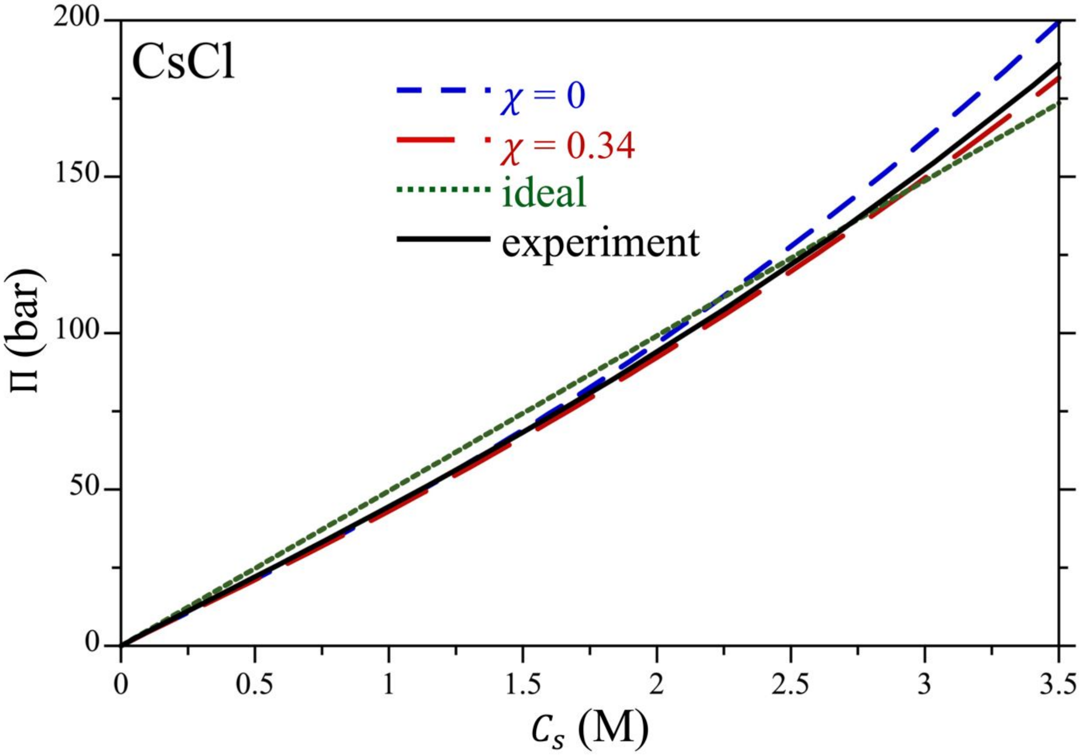

Figure S24. Osmotic pressure of CsCl in water as a function of the salt concentration. Results obtained from simulation using original ($\chi = 0$) and optimized ($\chi = 0.34$) potentials are compared against experiment and the ideal gas law. The experimental results are taken from Hammer and Wu.[1] The figure symbols are defined in the legend.

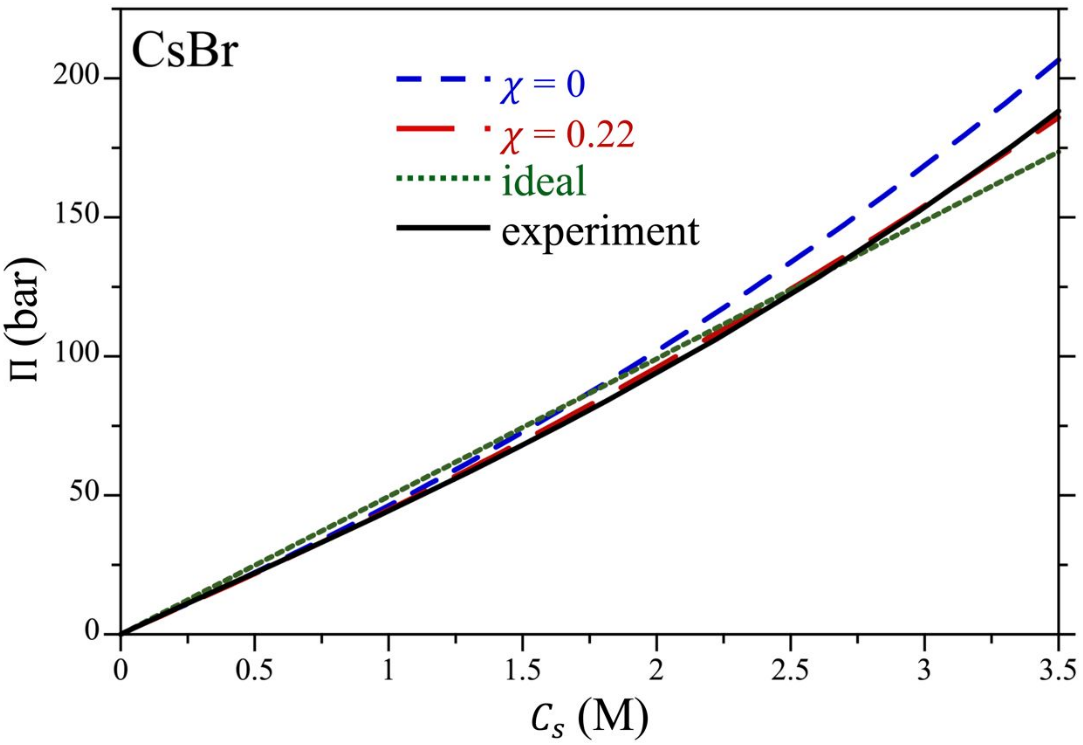

Figure S25. Osmotic pressure of CsBr in water as a function of the salt concentration. Results obtained from simulation using original ($\chi = 0$) and optimized ($\chi = 0.22$) potentials are compared against experiment and the ideal gas law. The experimental results are taken from Hammer and Wu.[1] The figure symbols are defined in the legend.

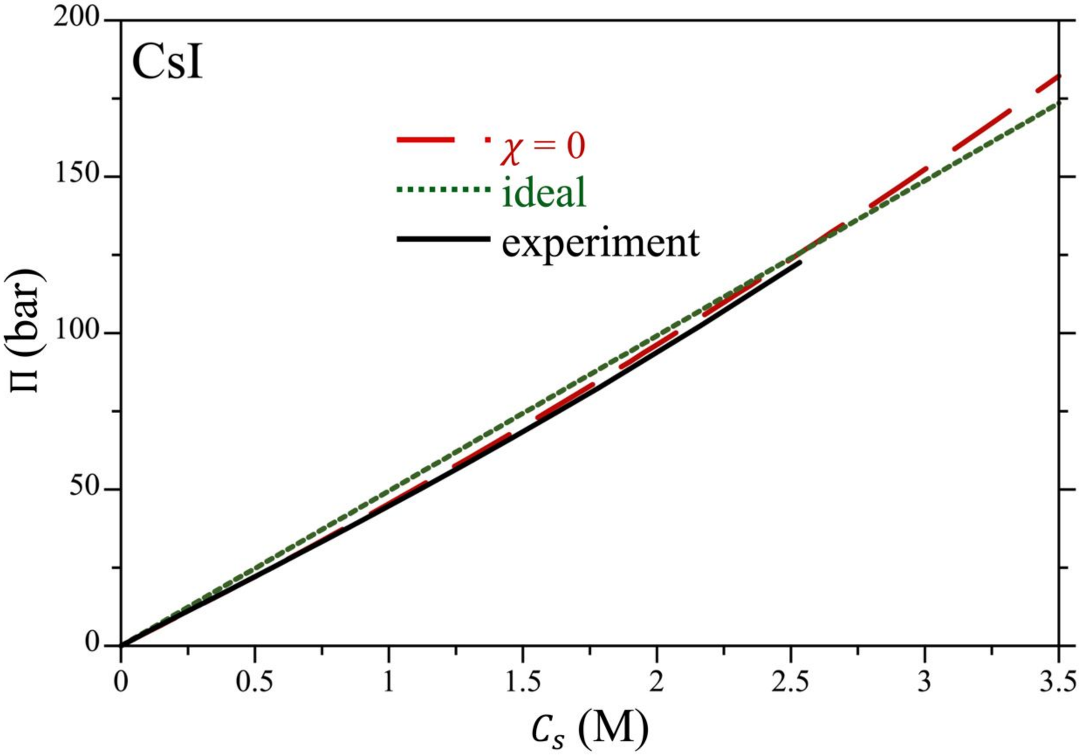

Figure S26. Osmotic pressure of CsI in water as a function of the salt concentration. Results obtained from simulation using the original/optimized ($\chi = 0$) potential are compared against experiment and the ideal gas law. The experimental results are taken from Hammer and Wu.[1] The figure symbols are defined in the legend.

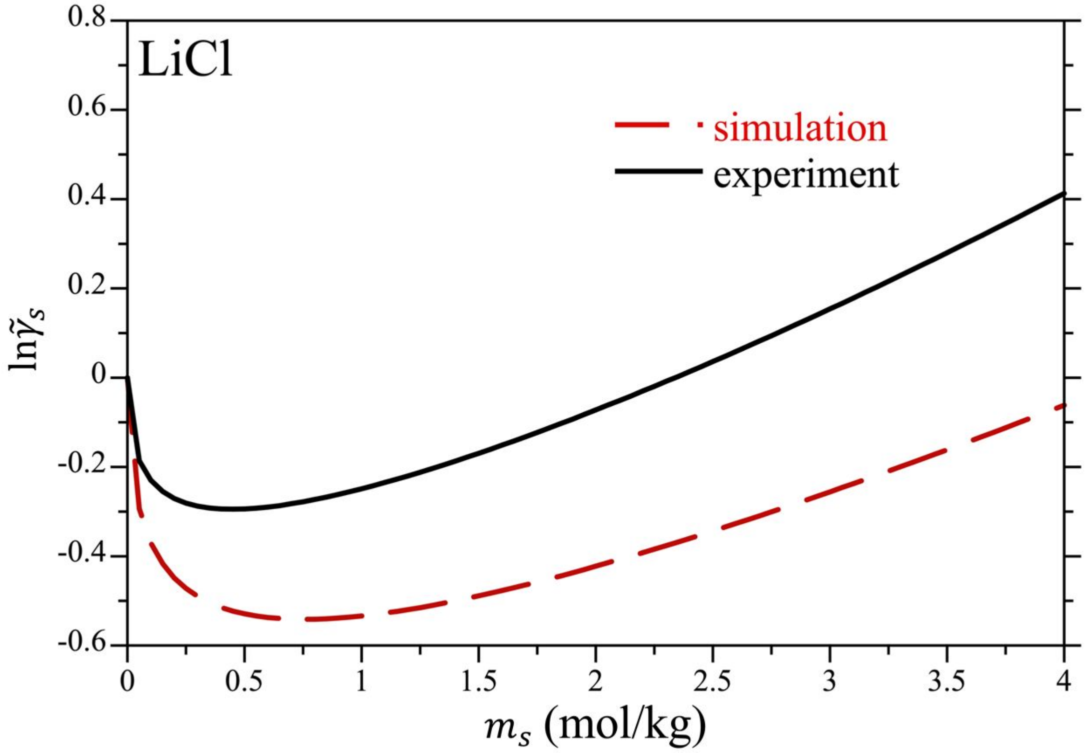

Figure S27. Activity coefficients of LiCl in water at ambient pressure by fitting eq. (20) to the osmotic pressures of the salts at 25°C. The simulations were conducted using the optimized Lennard-Jones cross interactions. The experimental results are taken from Hammer and Wu.[1] The figure symbols are defined in the legend.

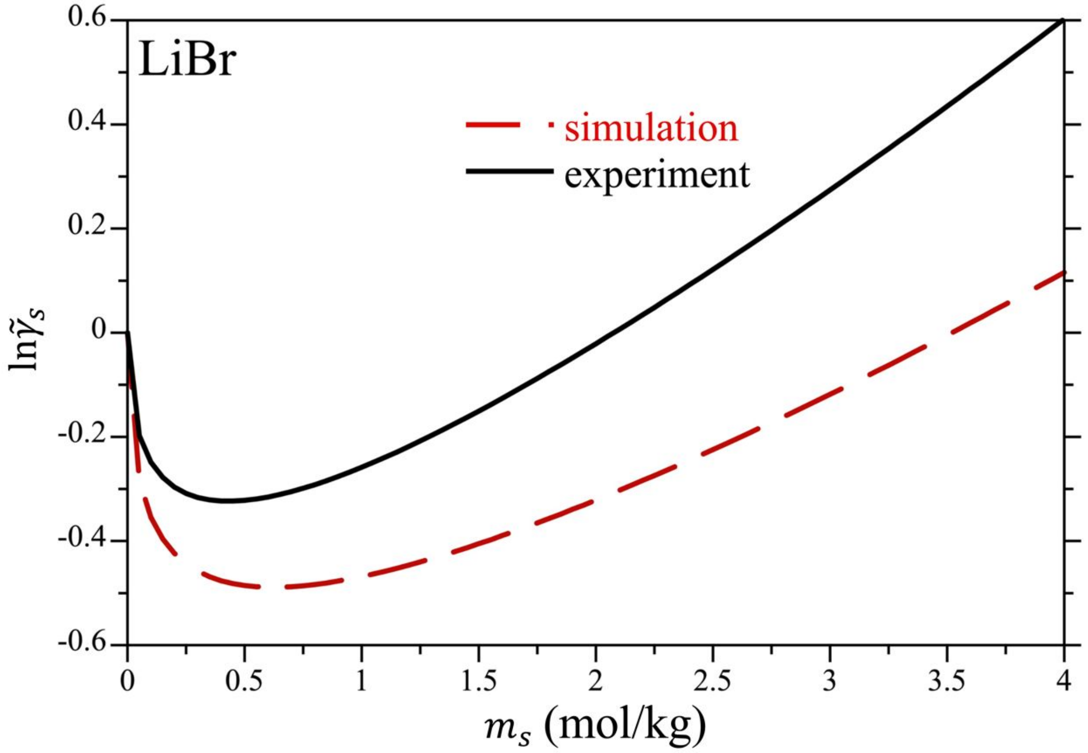

Figure S28. Activity coefficients of LiBr in water at ambient pressure by fitting eq. (20) to the osmotic pressures of the salts at 25°C. The simulations were conducted using the optimized Lennard-Jones cross interactions. The experimental results are taken from Hammer and Wu.[1] The figure symbols are defined in the legend.

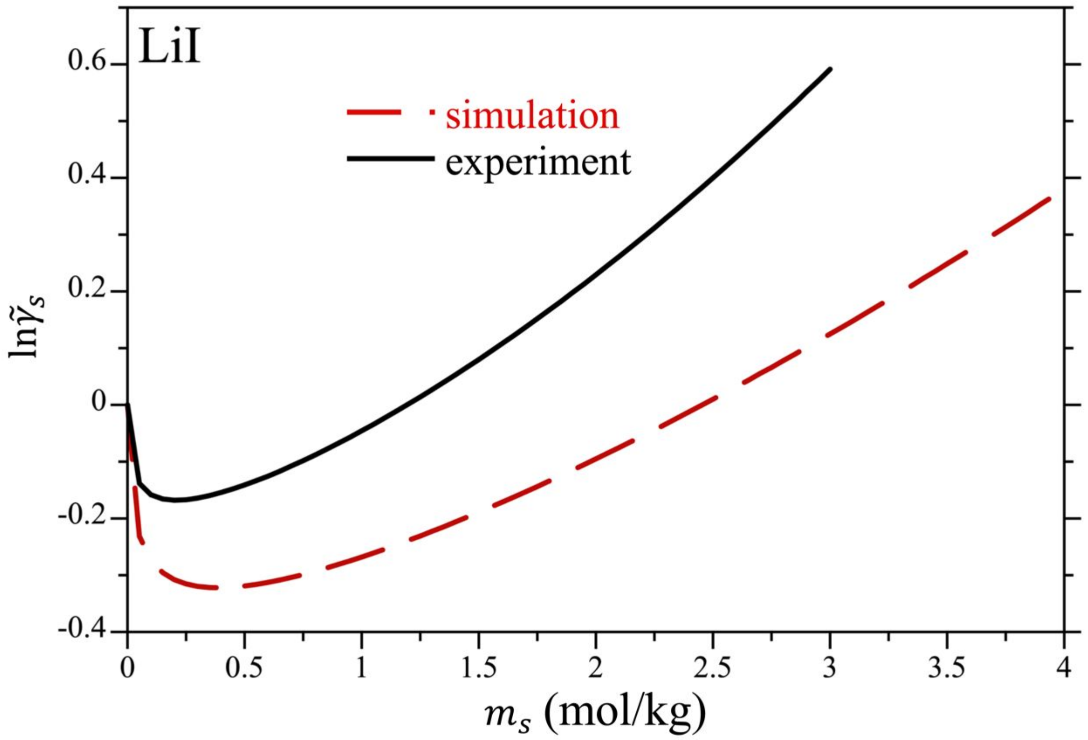

Figure S29. Activity coefficients of LiI in water at ambient pressure by fitting eq. (20) to the osmotic pressures of the salts at 25°C. The simulations were conducted using the optimized Lennard-Jones cross interactions. The experimental results are taken from Hammer and Wu.[1] The figure symbols are defined in the legend.

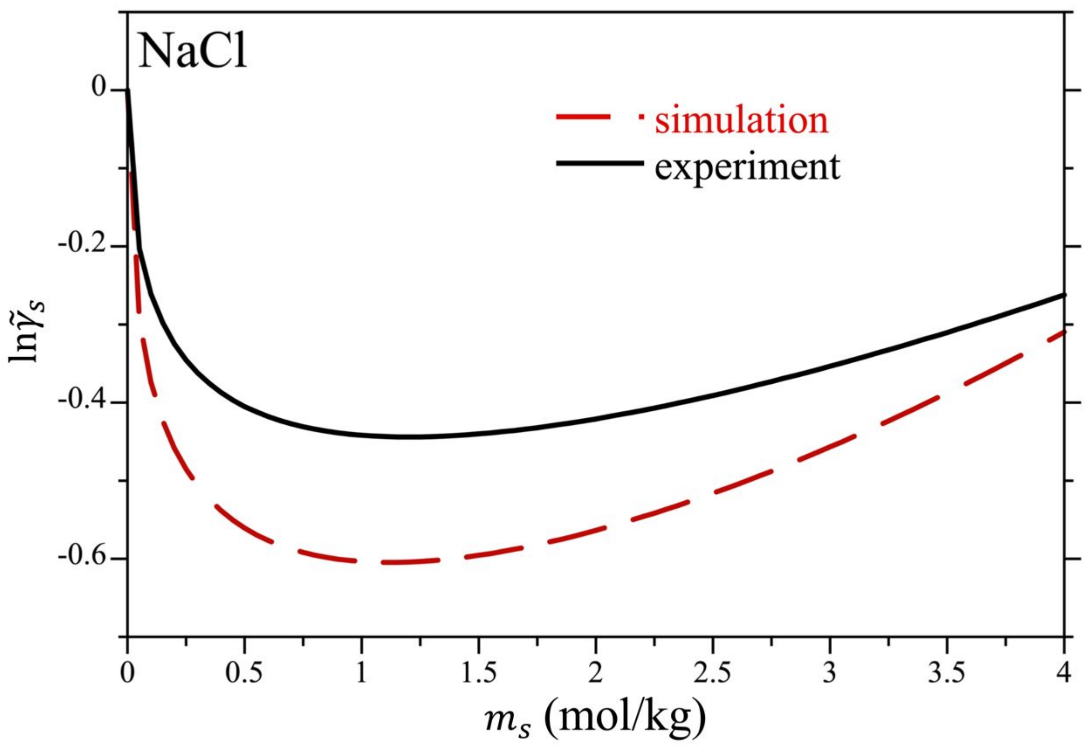

Figure S30. Activity coefficients of NaCl in water at ambient pressure by fitting eq. (20) to the osmotic pressures of the salts at 25°C. The simulations were conducted using the optimized Lennard-Jones cross interactions. The experimental results are taken from Hammer and Wu.[1] The figure symbols are defined in the legend.

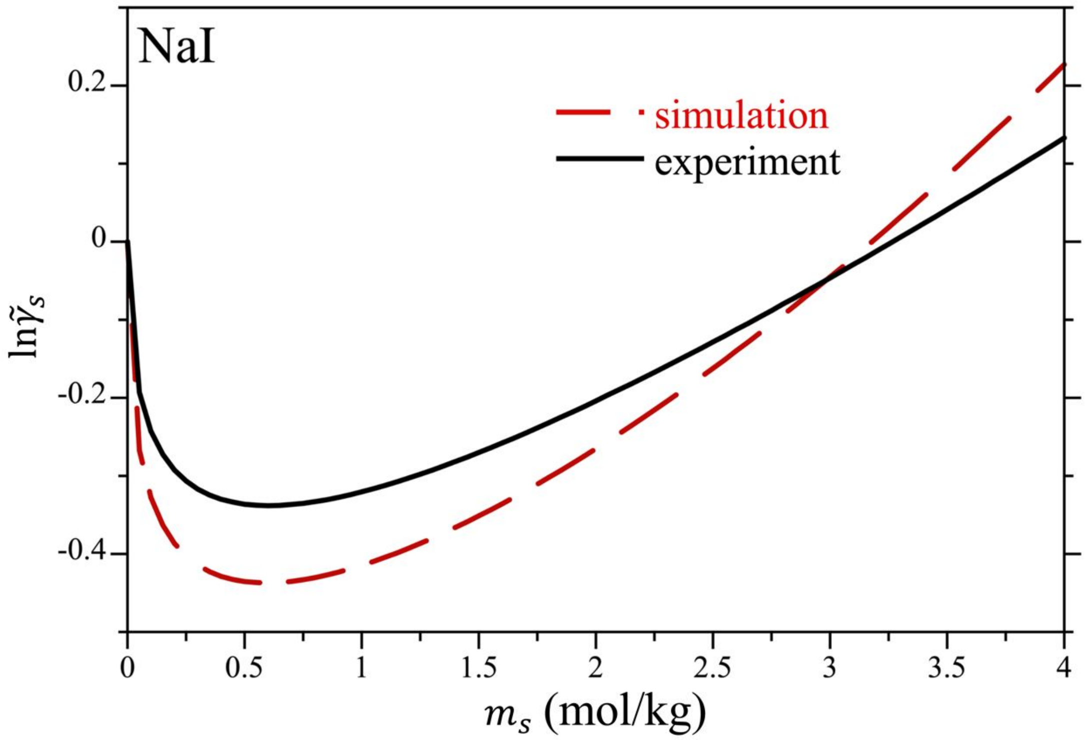

Figure S31. Activity coefficients of NaI in water at ambient pressure by fitting eq. (20) to the osmotic pressures of the salts at 25°C. The simulations were conducted using the optimized Lennard-Jones cross interactions. The experimental results are taken from Hammer and Wu.[1] The figure symbols are defined in the legend.

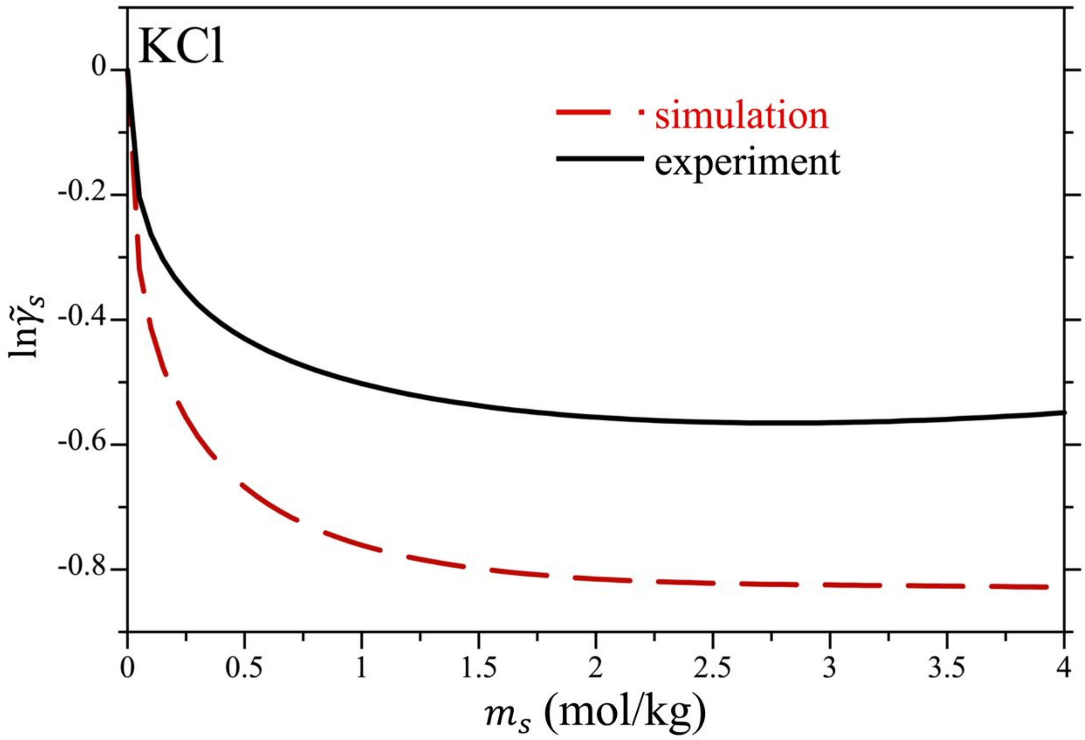

Figure S32. Activity coefficients of KCl in water at ambient pressure by fitting eq. (20) to the osmotic pressures of the salts at 25°C. The simulations were conducted using the optimized Lennard-Jones cross interactions. The experimental results are taken from Hammer and Wu.[1] The figure symbols are defined in the legend.

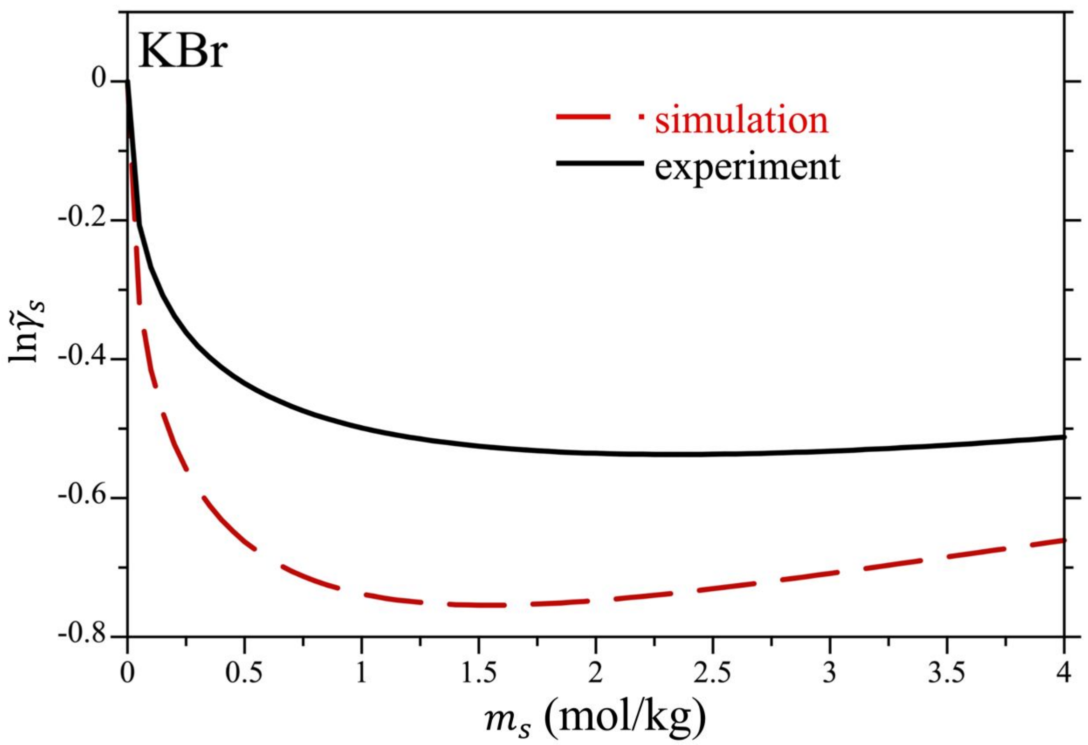

Figure S33. Activity coefficients of KBr in water at ambient pressure by fitting eq. (20) to the osmotic pressures of the salts at 25°C. The simulations were conducted using the optimized Lennard-Jones cross interactions. The experimental results are taken from Hammer and Wu.[1] The figure symbols are defined in the legend.

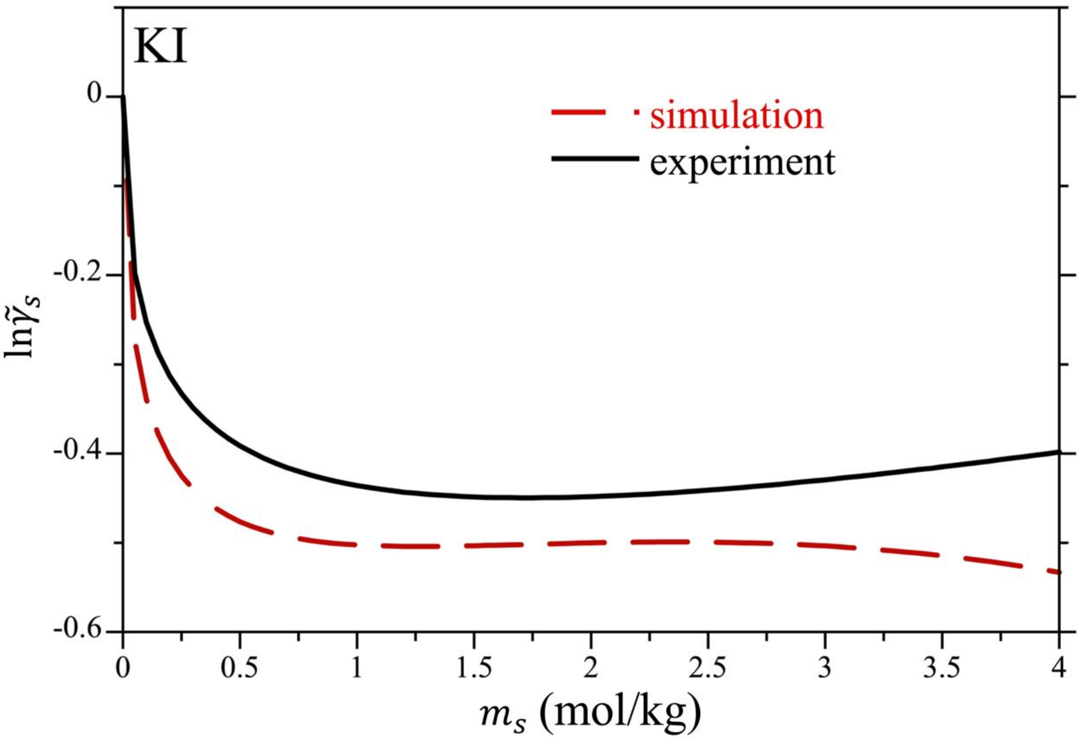

Figure S34. Activity coefficients of KI in water at ambient pressure by fitting eq. (20) to the osmotic pressures of the salts at 25°C. The simulations were conducted using the optimized Lennard-Jones cross interactions. The experimental results are taken from Hammer and Wu.[1] The figure symbols are defined in the legend.

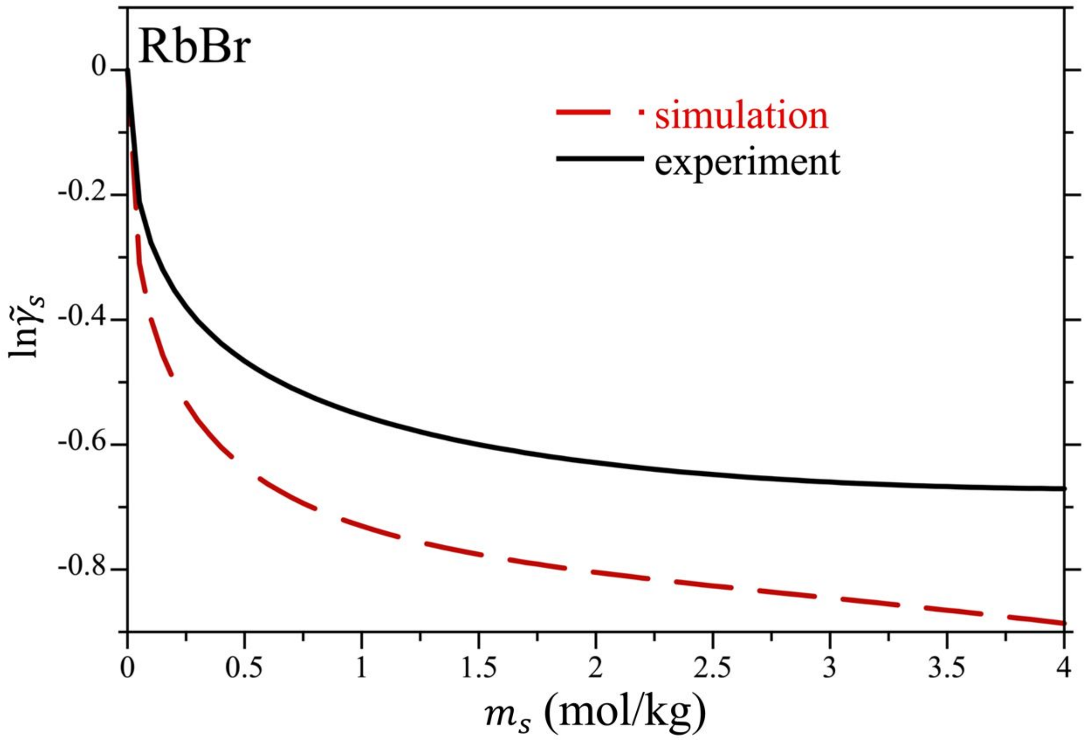

Figure S35. Activity coefficients of RbBr in water at ambient pressure by fitting eq. (20) to the osmotic pressures of the salts at 25°C. The simulations were conducted using the optimized Lennard-Jones cross interactions. The experimental results are taken from Hammer and Wu.[1] The figure symbols are defined in the legend.

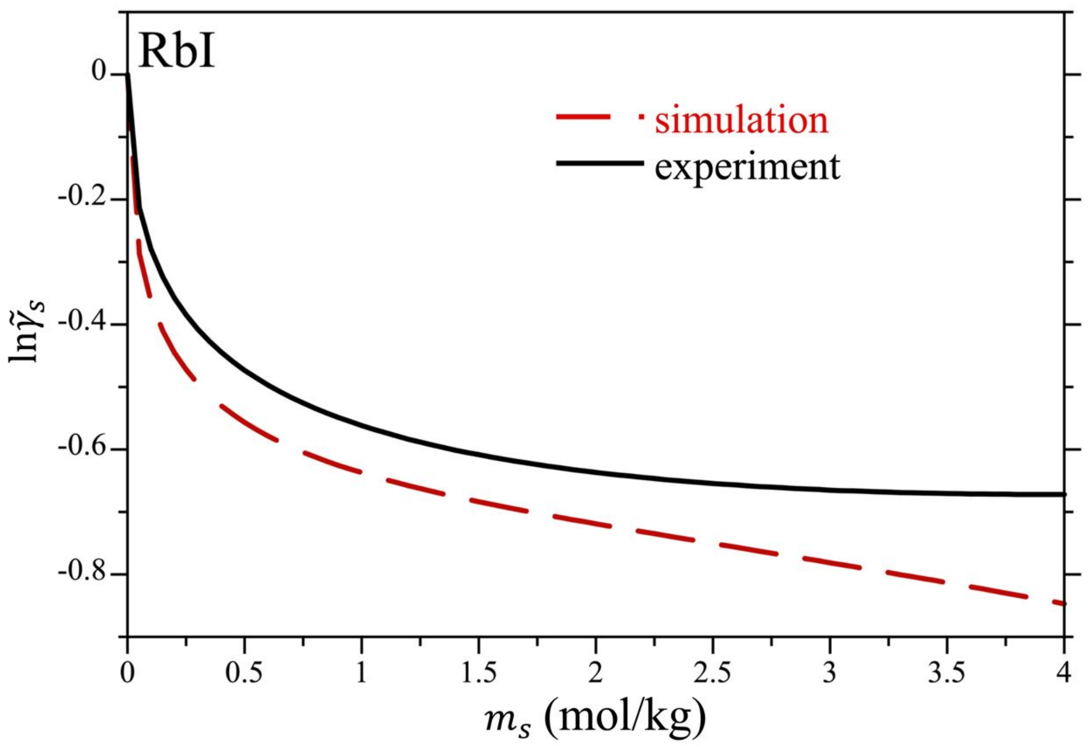

Figure S36. Activity coefficients of RbI in water at ambient pressure by fitting eq. (20) to the osmotic pressures of the salts at 25°C. The simulations were conducted using the optimized Lennard-Jones cross interactions. The experimental results are taken from Hammer and Wu.[1] The figure symbols are defined in the legend.

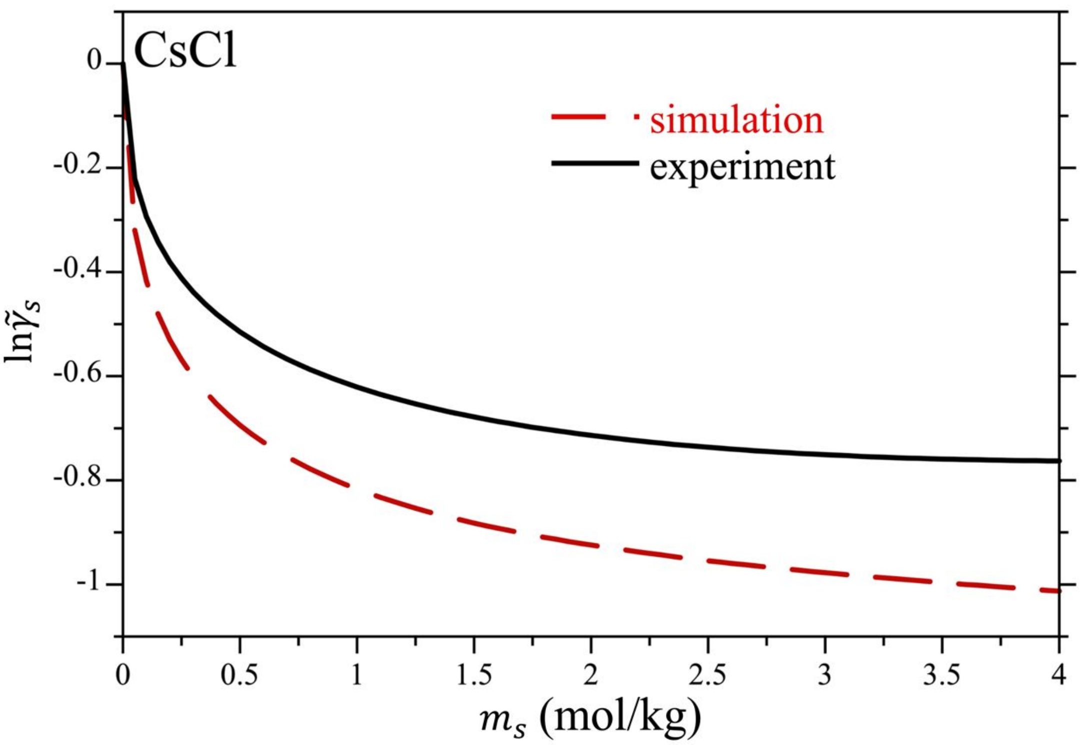

Figure S37. Activity coefficients of CsCl in water at ambient pressure by fitting eq. (20) to the osmotic pressures of the salts at 25°C. The simulations were conducted using the optimized Lennard-Jones cross interactions. The experimental results are taken from Hammer and Wu.[1] The figure symbols are defined in the legend.

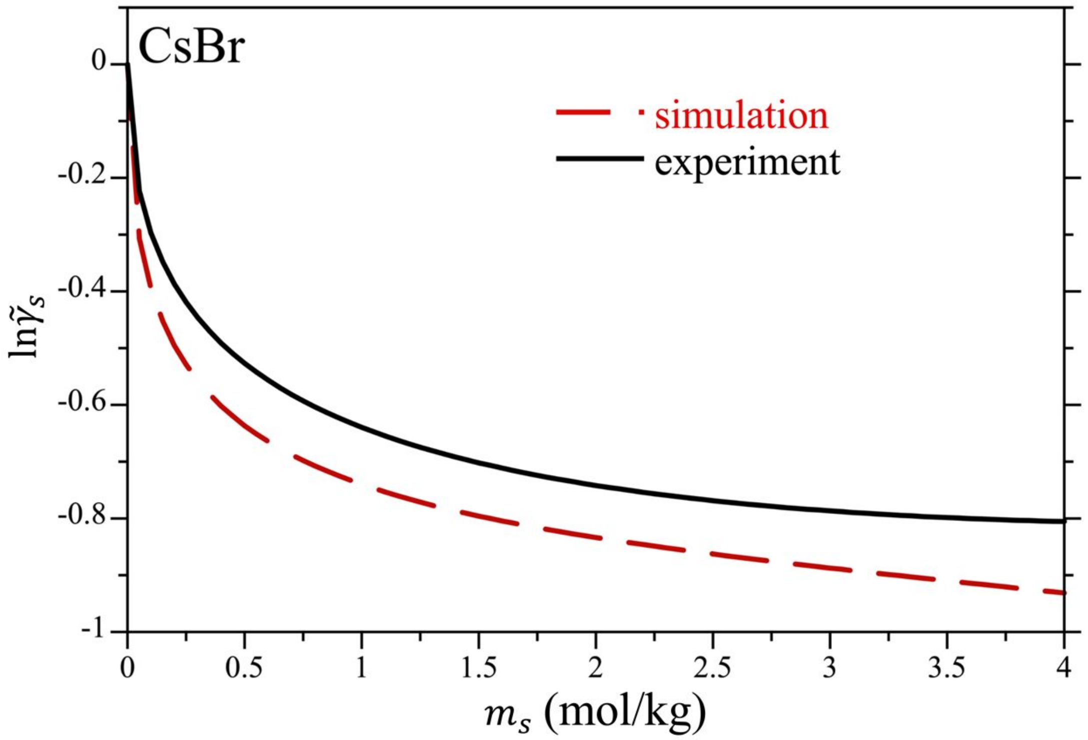

Figure S38. Activity coefficients of CsBr in water at ambient pressure by fitting eq. (20) to the osmotic pressures of the salts at 25°C. The simulations were conducted using the optimized Lennard-Jones cross interactions. The experimental results are taken from Hammer and Wu.[1] The figure symbols are defined in the legend.

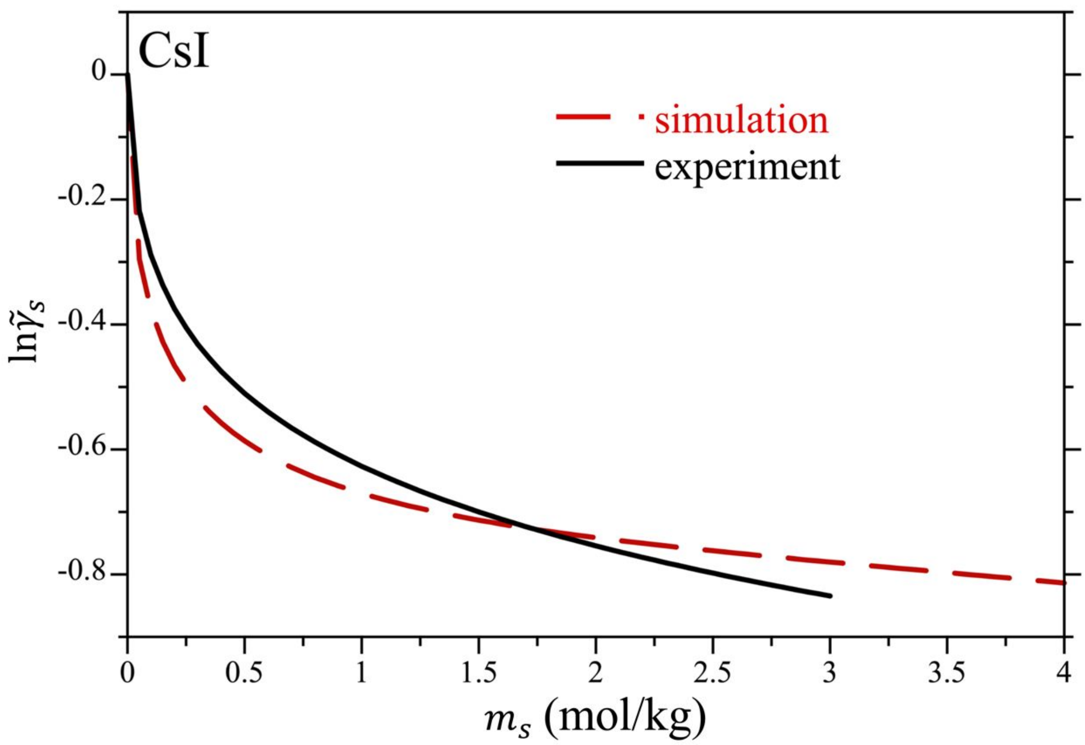

Figure S39. Activity coefficients of CsI in water at ambient pressure by fitting eq. (20) to the osmotic pressures of the salts at 25°C. The simulations were conducted using the optimized Lennard-Jones cross interactions. The experimental results are taken from Hammer and Wu.[1] The figure symbols are defined in the legend.

References.

(1) Hammer, W. J.; Wu, Y. C. Osmotic Coefficients and Mean Activity Coefficients of Uni-Univalent Electrolytes in Water at 25°C. *Journal of Physical and Chemical Reference Data* **1972**, *1*, 1047-1100.
